# Supplementary figures and images for: Cell-free DNA as a potential biomarker of differentiation and toxicity in cardiac organoids
Source: eLife. 2023 Jun 1;12:e83532. doi: 10.7554/eLife.83532 (PMC10287154; doi:10.7554/eLife.83532)

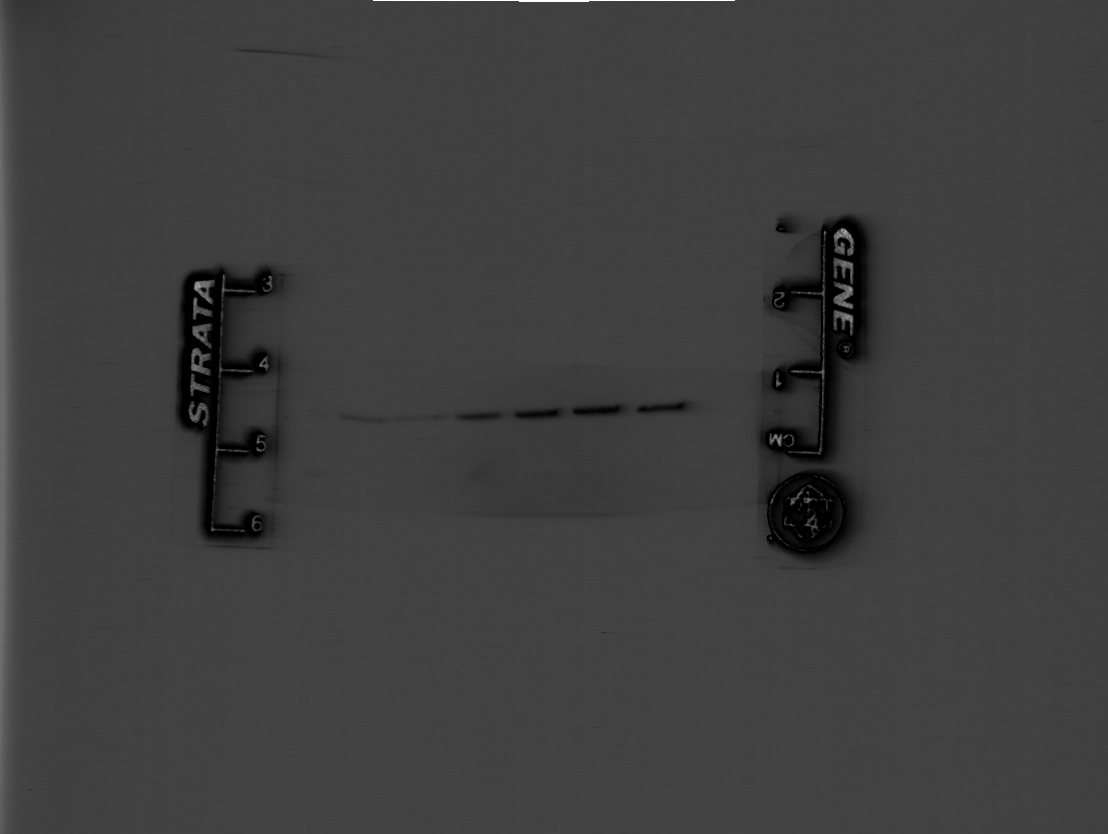

Supplement: Figure 1—source data 1. [file elife-83532-fig1-data1.zip › Figure 1 - source data 1/Western Blot _figure 1_ uncropped, labeled and unlabeled/Unedited alpha actinin 10s exp.tif]

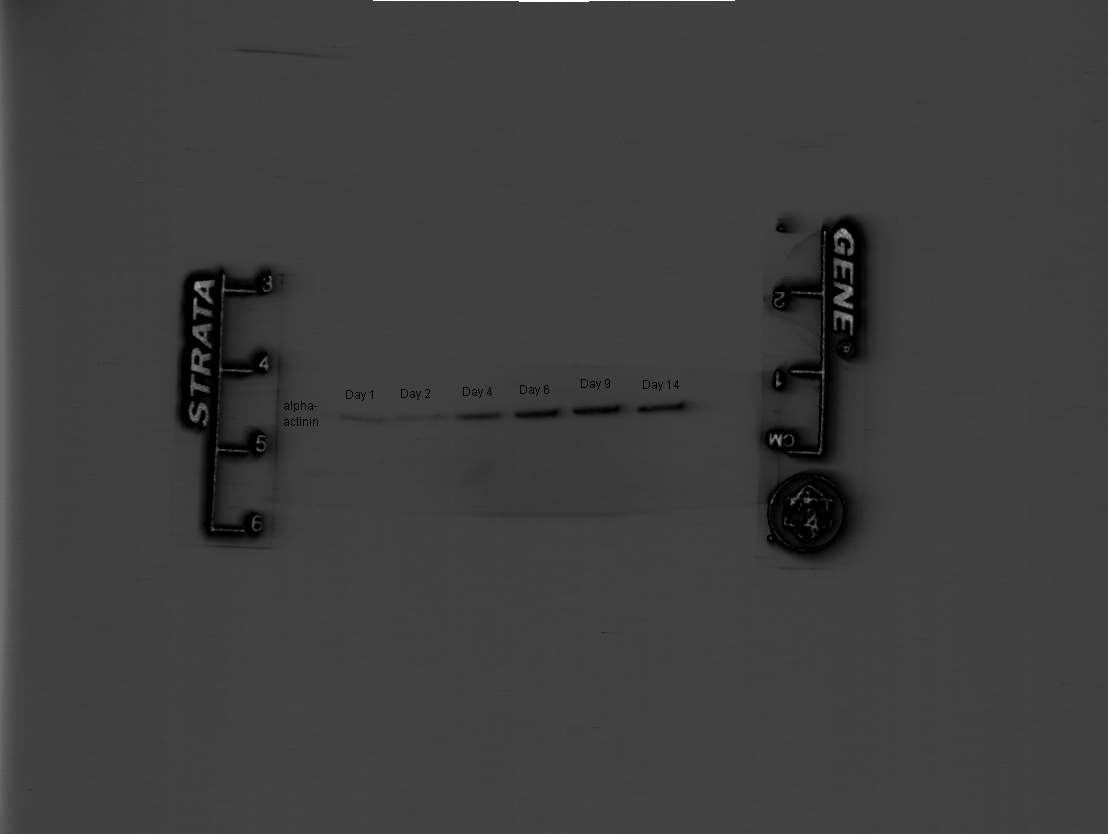

Supplement: Figure 1—source data 1. [file elife-83532-fig1-data1.zip › Figure 1 - source data 1/Western Blot _figure 1_ uncropped, labeled and unlabeled/Unedited alpha actinin labelled.tif]

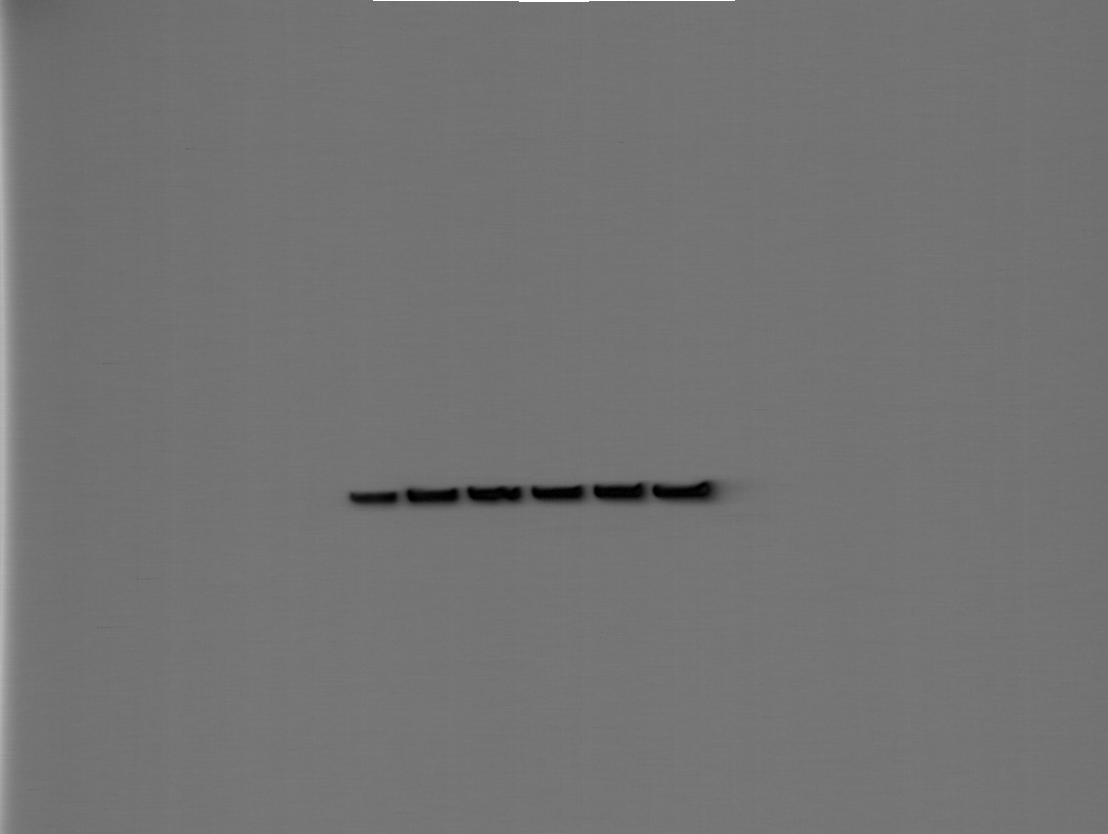

Supplement: Figure 1—source data 1. [file elife-83532-fig1-data1.zip › Figure 1 - source data 1/Western Blot _figure 1_ uncropped, labeled and unlabeled/Unedited Gapdh 4sec exp.tif]

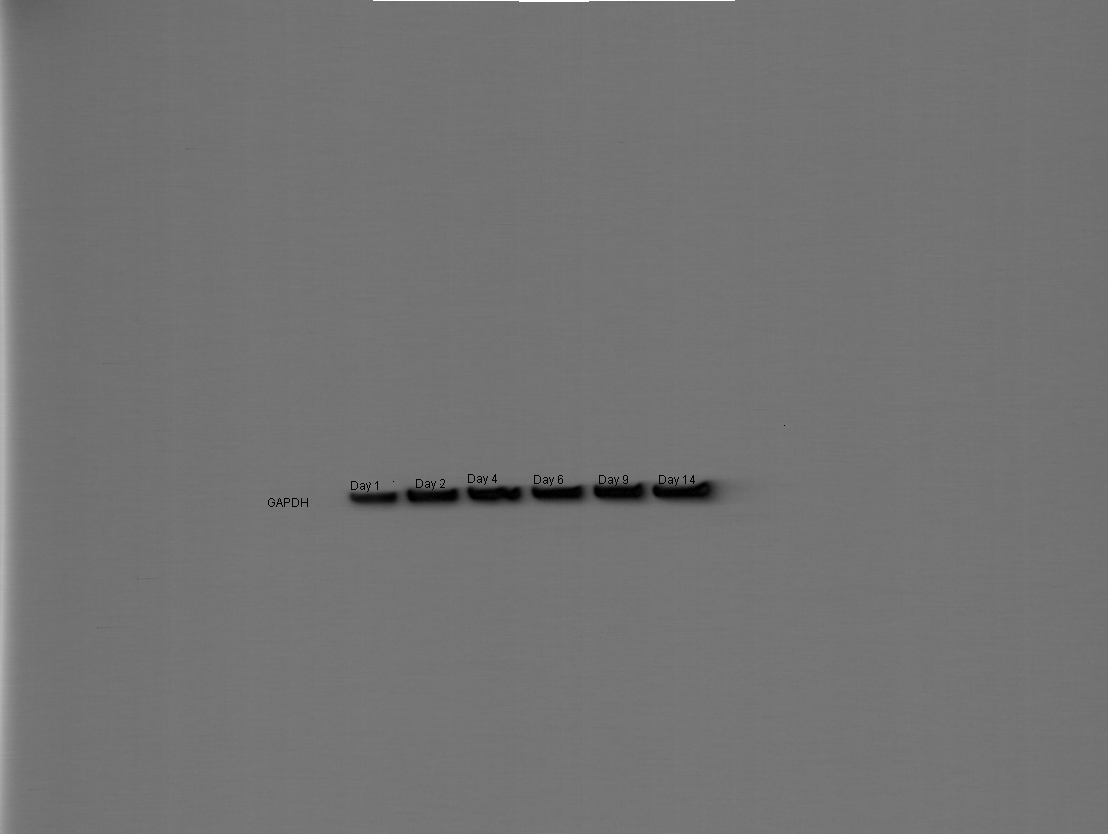

Supplement: Figure 1—source data 1. [file elife-83532-fig1-data1.zip › Figure 1 - source data 1/Western Blot _figure 1_ uncropped, labeled and unlabeled/Unedited Gapdh_labelled .tif]

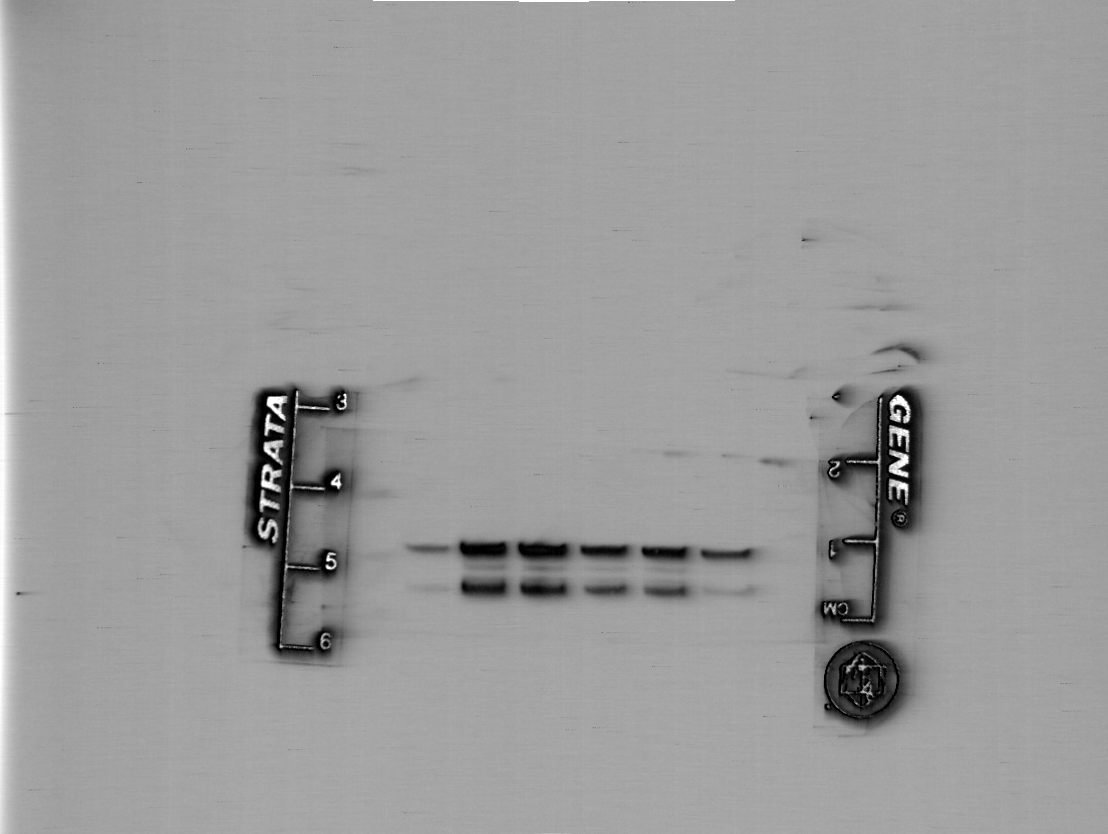

Supplement: Figure 1—source data 1. [file elife-83532-fig1-data1.zip › Figure 1 - source data 1/Western Blot _figure 1_ uncropped, labeled and unlabeled/Unedited gata6 30sec exp.tif]

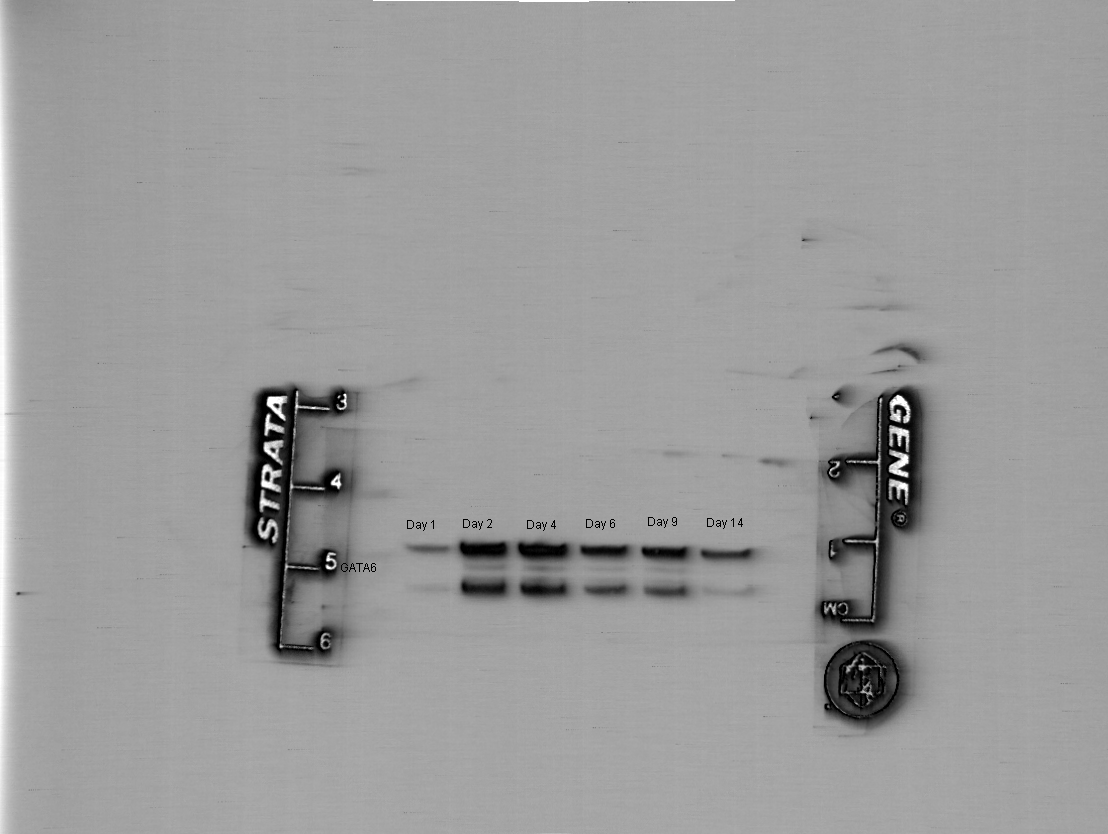

Supplement: Figure 1—source data 1. [file elife-83532-fig1-data1.zip › Figure 1 - source data 1/Western Blot _figure 1_ uncropped, labeled and unlabeled/Unedited gata6 labelled.tif]

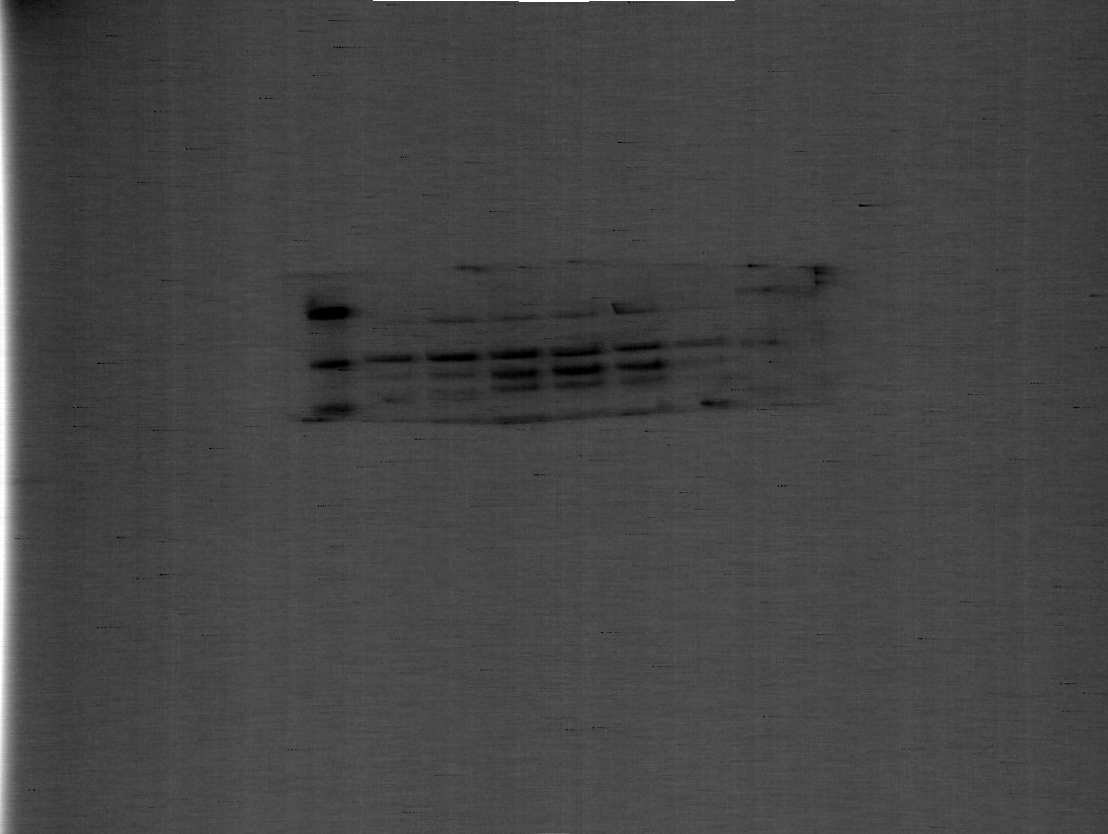

Supplement: Figure 1—source data 1. [file elife-83532-fig1-data1.zip › Figure 1 - source data 1/Western Blot _figure 1_ uncropped, labeled and unlabeled/unedited mef2c 30s exp.tif]

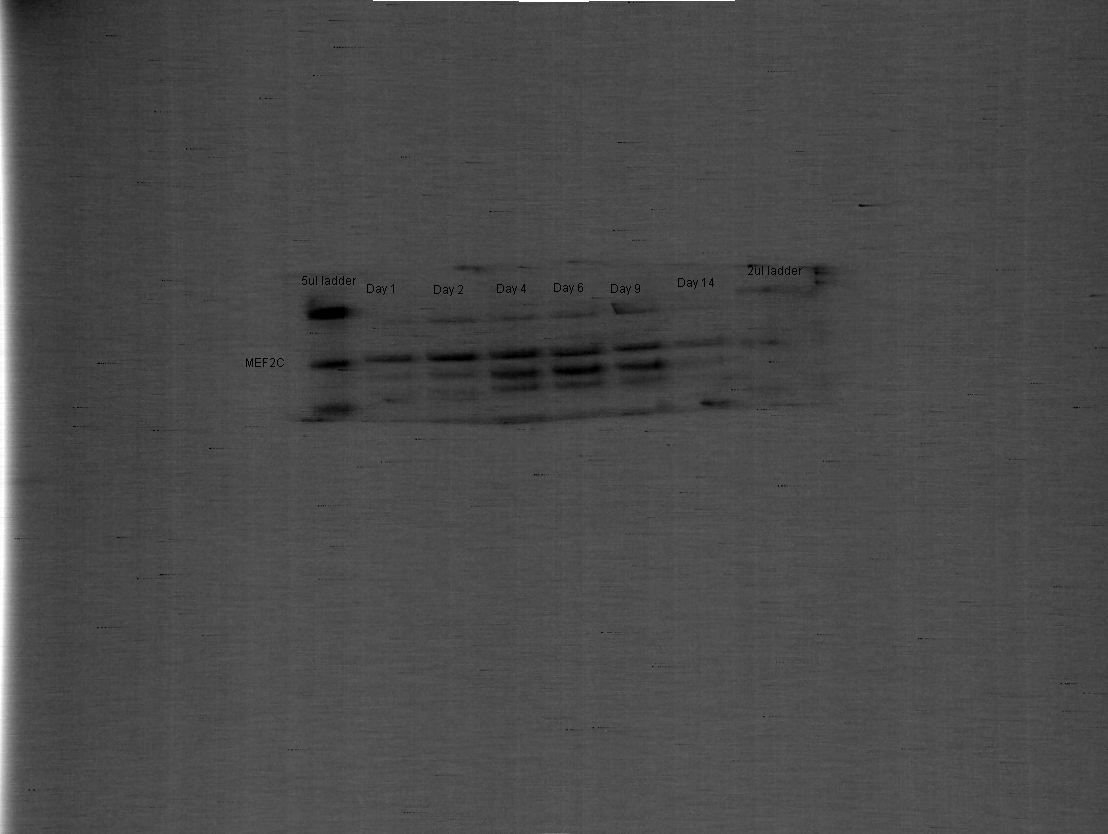

Supplement: Figure 1—source data 1. [file elife-83532-fig1-data1.zip › Figure 1 - source data 1/Western Blot _figure 1_ uncropped, labeled and unlabeled/unedited mef2c labelled.tif]

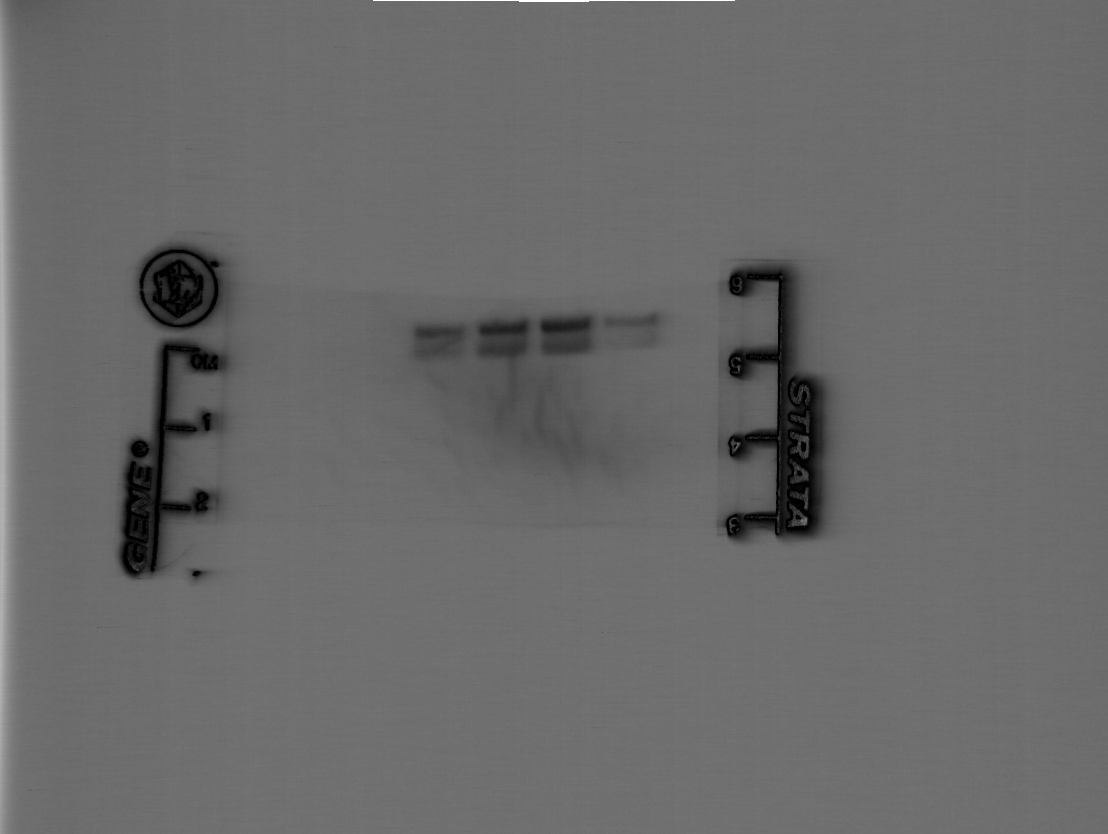

Supplement: Figure 1—source data 1. [file elife-83532-fig1-data1.zip › Figure 1 - source data 1/Western Blot _figure 1_ uncropped, labeled and unlabeled/unedited nkx2-5 5s exp.tif]

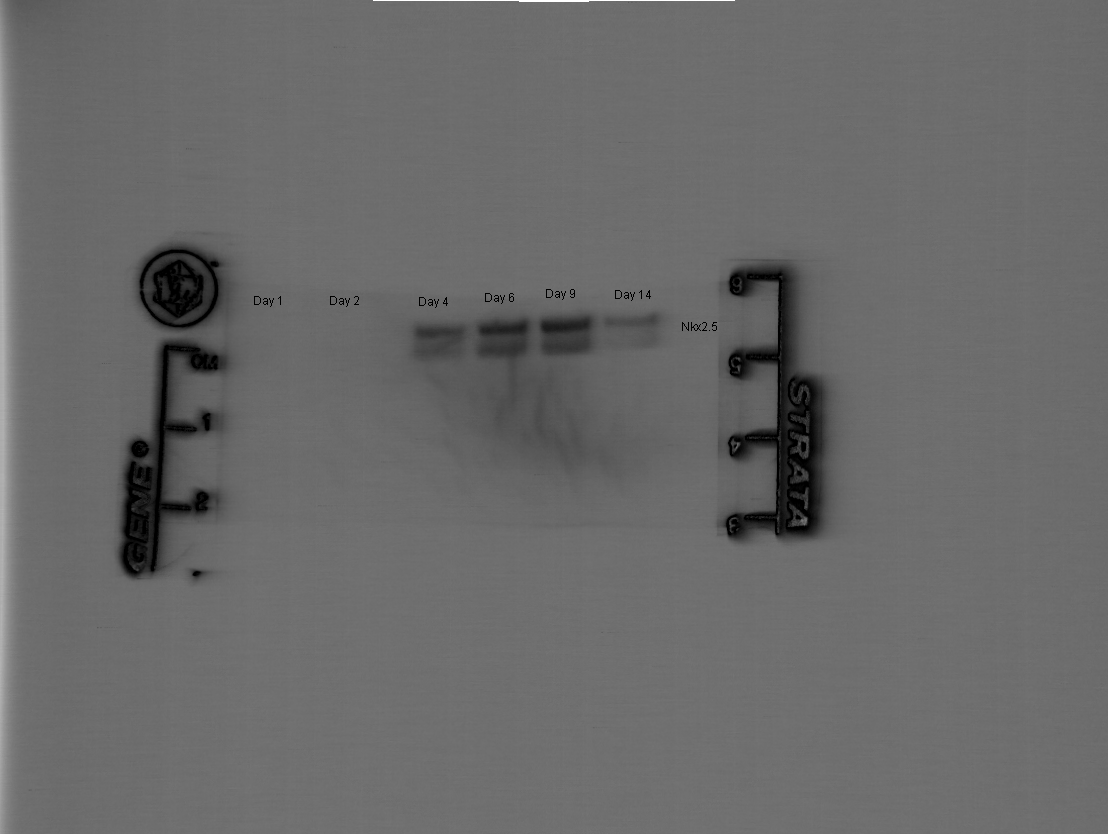

Supplement: Figure 1—source data 1. [file elife-83532-fig1-data1.zip › Figure 1 - source data 1/Western Blot _figure 1_ uncropped, labeled and unlabeled/unedited nkx2-5 labelled.tif]

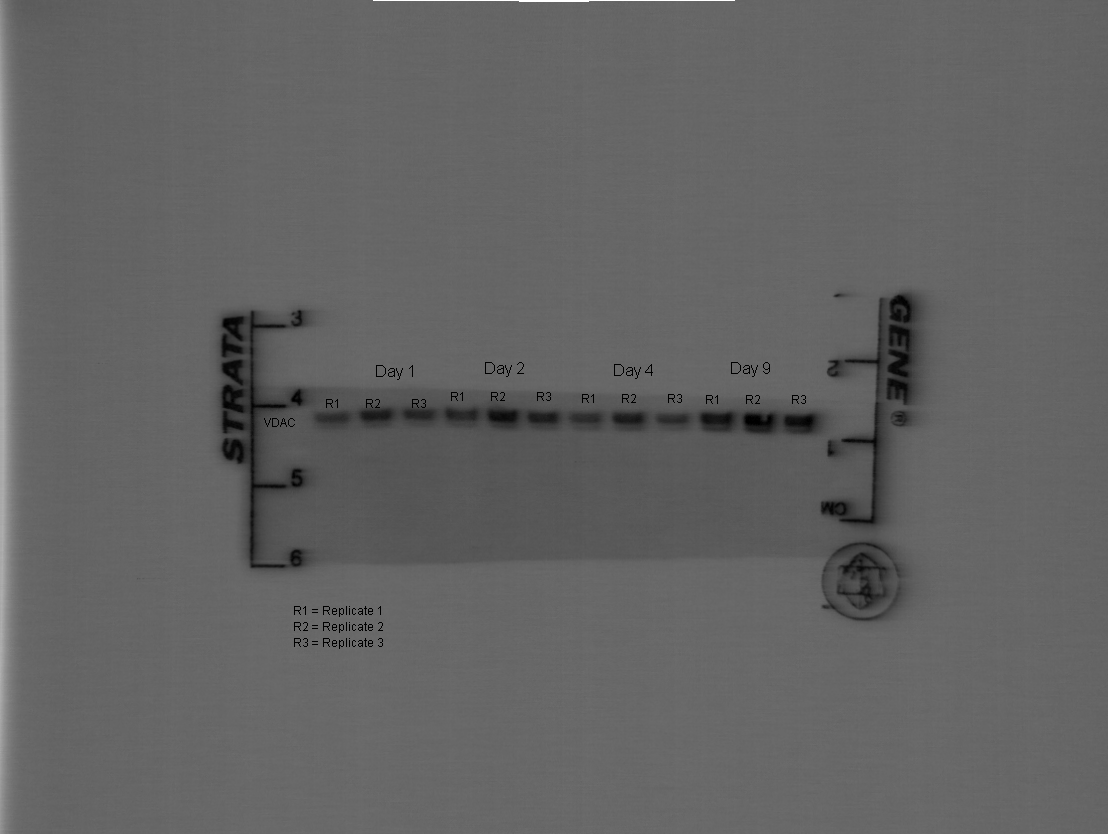

Supplement: Figure 3—source data 1. [file elife-83532-fig3-data1.zip › Figure 3 - source data 1/Western blot_figure 3E uncropped, labeled and unlabeled/unedited vdac 1 labelled.tif]

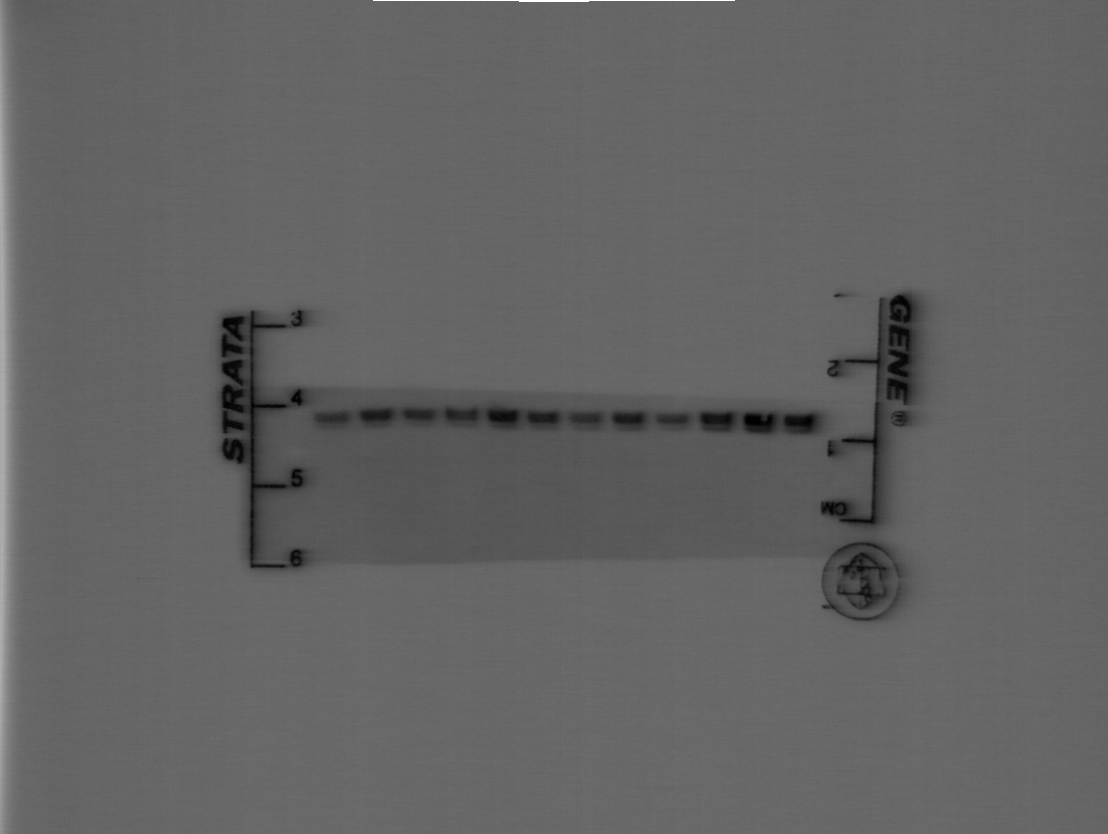

Supplement: Figure 3—source data 1. [file elife-83532-fig3-data1.zip › Figure 3 - source data 1/Western blot_figure 3E uncropped, labeled and unlabeled/unedited vdac 1 sec exposure.tif]

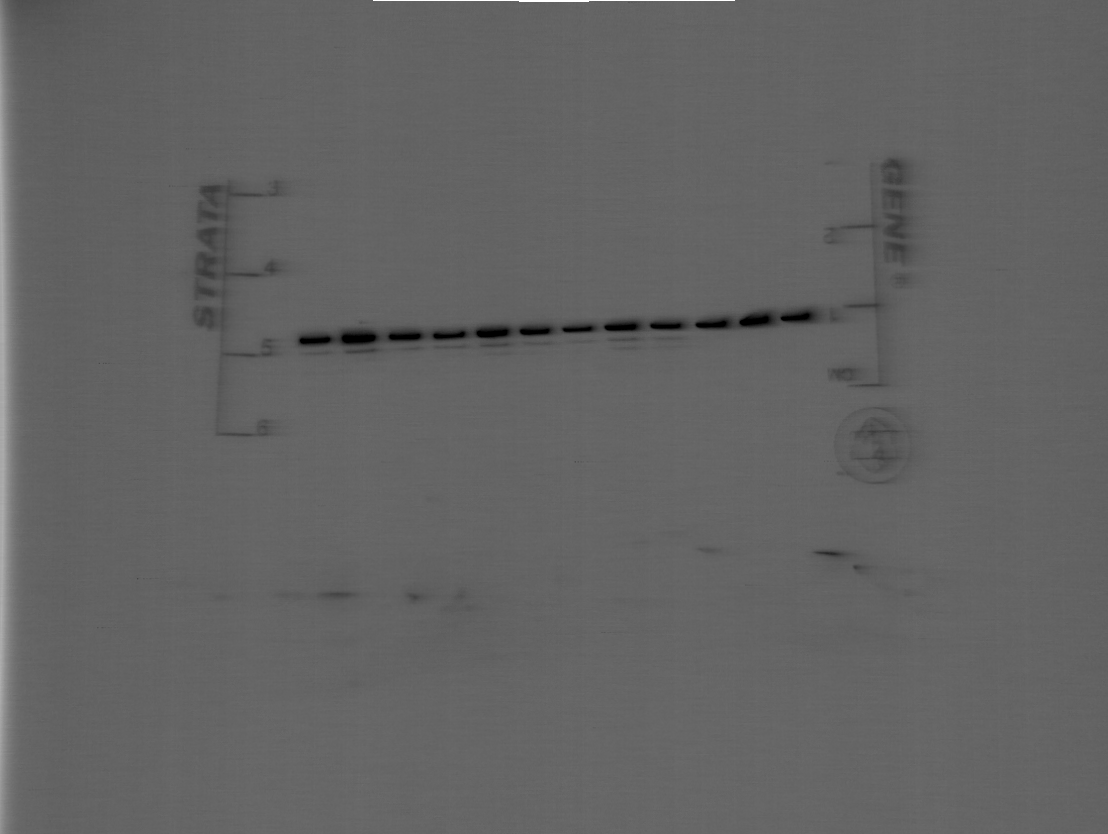

Supplement: Figure 3—source data 1. [file elife-83532-fig3-data1.zip › Figure 3 - source data 1/Western blot_figure 3E uncropped, labeled and unlabeled/Unedited vinculin, 5s exp .tif]

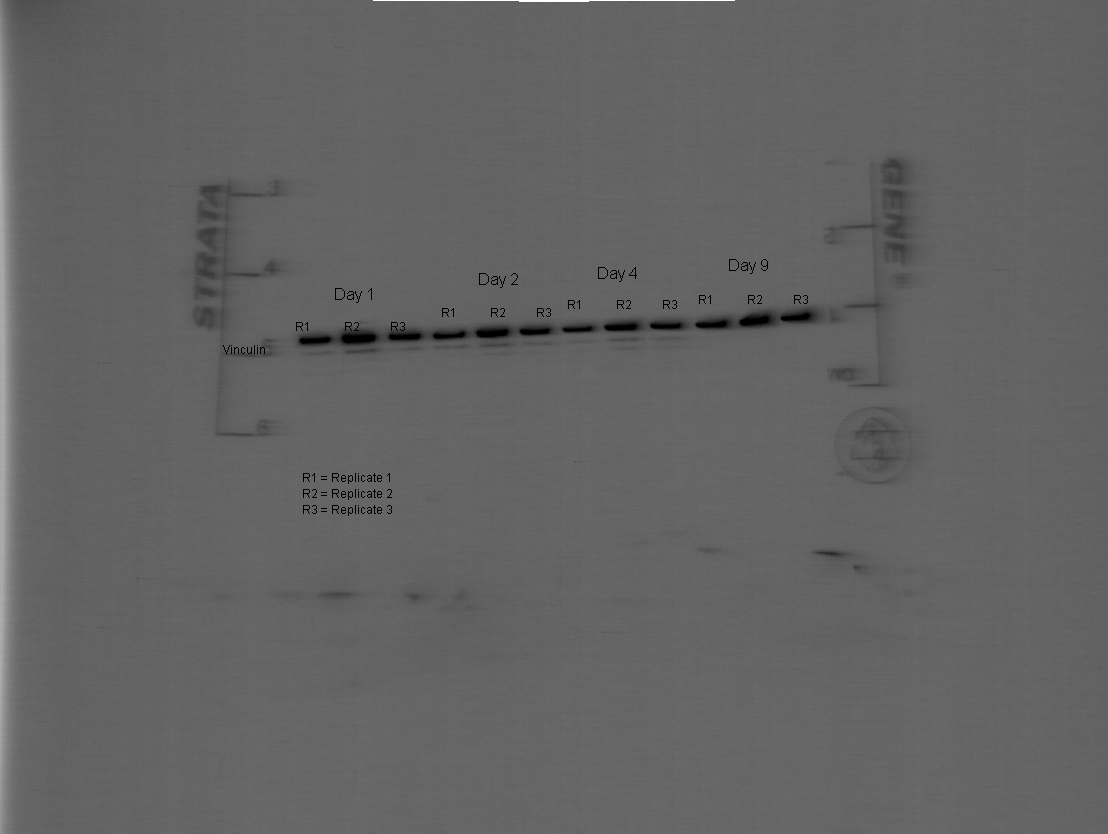

Supplement: Figure 3—source data 1. [file elife-83532-fig3-data1.zip › Figure 3 - source data 1/Western blot_figure 3E uncropped, labeled and unlabeled/Unedited vinculin, labelled .tif]

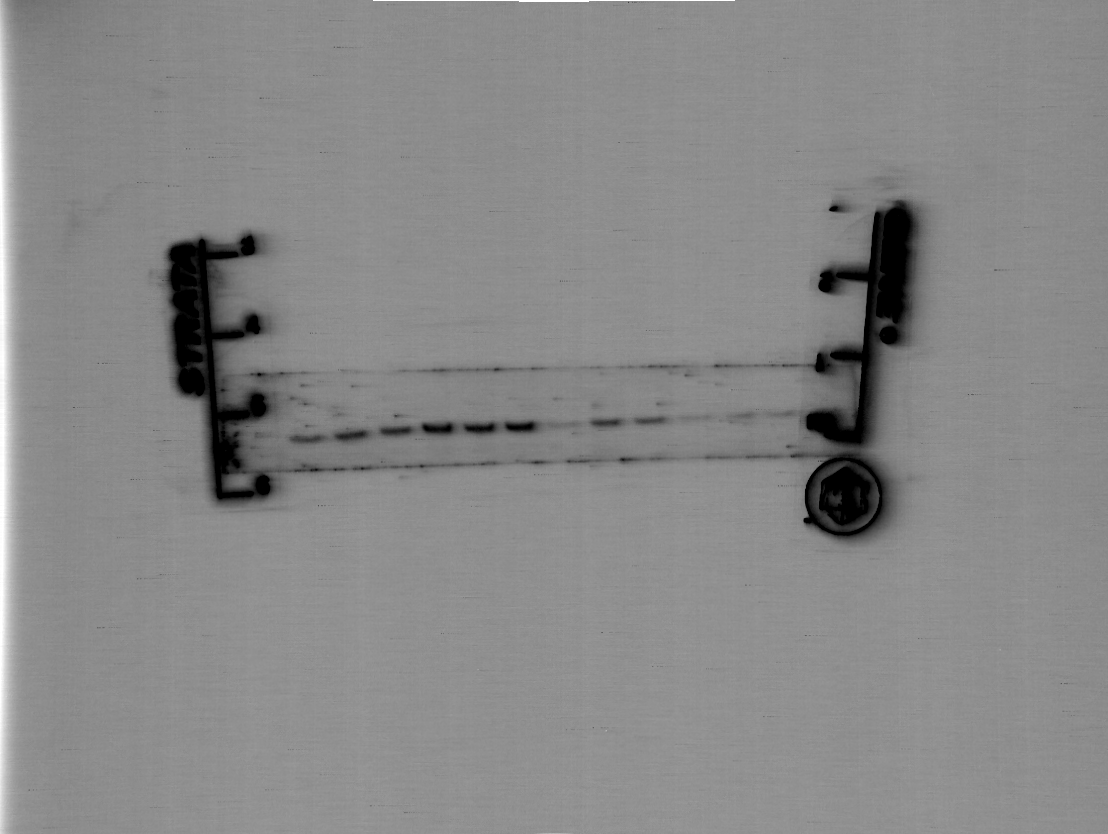

Supplement: Figure 4—source data 1. [file elife-83532-fig4-data1.zip › Figure 4 - source data 1/Western blot_figure 4D_uncropped, labeled and unlabeled/Unedited p53 45 sec.tif]

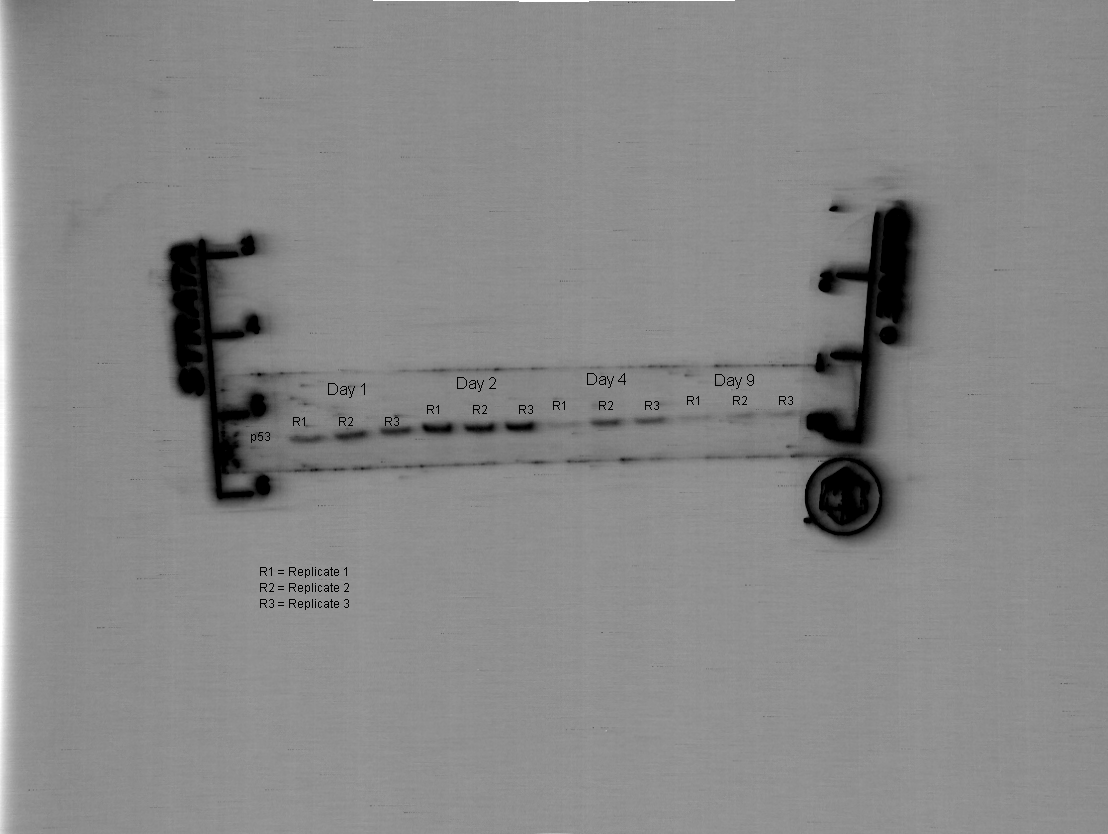

Supplement: Figure 4—source data 1. [file elife-83532-fig4-data1.zip › Figure 4 - source data 1/Western blot_figure 4D_uncropped, labeled and unlabeled/Unedited p53 Labelled.tif]
