# Supplementary material for: Cell-free DNA as a potential biomarker of differentiation and toxicity in cardiac organoids
Source: eLife. 2023 Jun 1;12:e83532. doi: 10.7554/eLife.83532 (PMC10287154; doi:10.7554/eLife.83532)
Supplement: Figure 2—source data 1. [file elife-83532-fig2-data1.zip › Figure 2 - source data/Figure 2D_raw_fragment analyzer data days 6, 7, 9, 12 cardiac cfDNA.pdf]

Fragment Analyzer Run Summary:

Filename and Data Path: C:\AATI\Data\2021 07 21\17-10-29\2021 07 21 17H 10M.raw
Created: Wednesday, July 21, 2021 5:26:30 PM
# of Capillaries: 12
Array Serial #: 072512-05LFS
Effect Length: 55 cm
Array Usage Count: 1817
FA Version #: 1.0.2.9
Device Serial #: 2656

METHOD INFORMATION
Method Name: DNF-464-55 - HS Ext Large Fragment.mthds
Gel Prime: No
Full Conditioning: Yes
Gel Prime to Buffer: No
Gel Selection: Gel 1
Perform Prerun: 7.0 kV, 30 sec.
Rinse: No
Marker 1: No
Rinse: Tray: 3, Row: A, # Dips: 1
Sample Injection: 9.0 kV, 30 sec.
Separation: 7.0 kV, 95.0 min.
Tray Name: Tray-1

Analysis Mode: NGS

NOTE

Copyright 2015 Advanced Analytical Technologies, Inc.

Gel Image

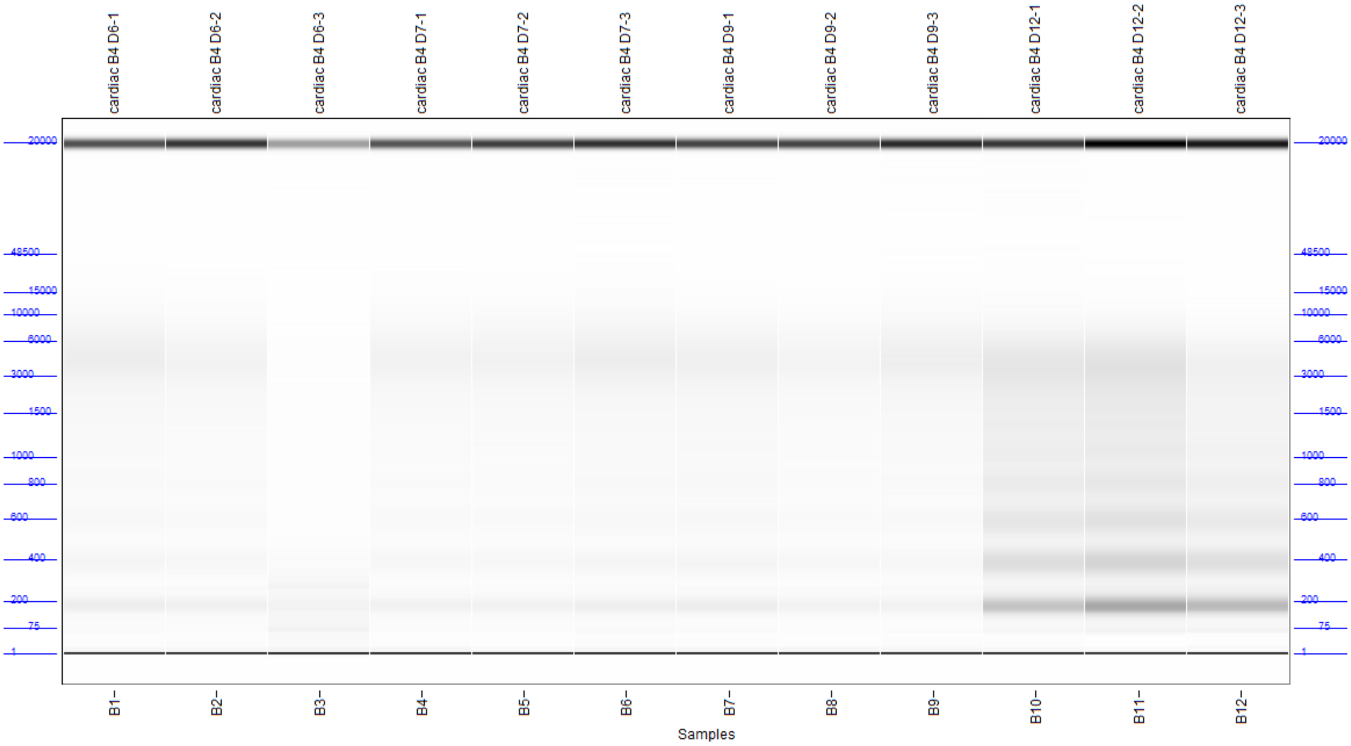

Copyright 2015 Advanced Analytical Technologies, Inc.

Filename and Data Path: C:\AATI\Data\2021 07 21\17-10-29\2021 07 21 17H 10M.raw

B1: cardiac B4 D6-1

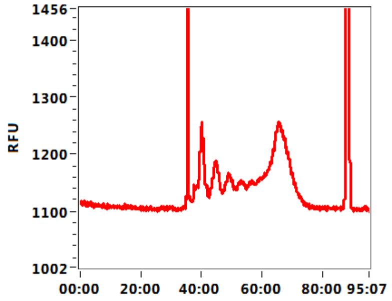

B2: cardiac B4 D6-2

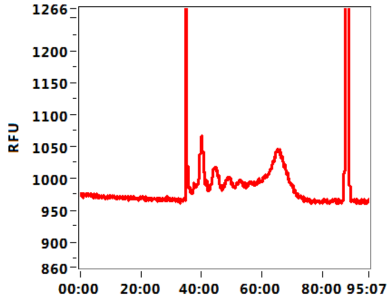

B3: cardiac B4 D6-3

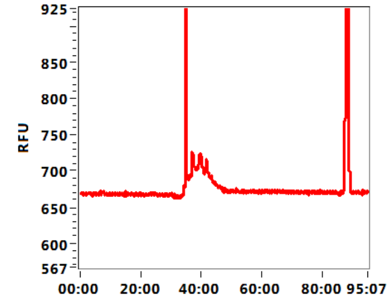

B4: cardiac B4 D7-1

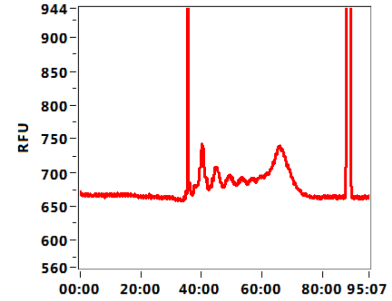

B5: cardiac B4 D7-2

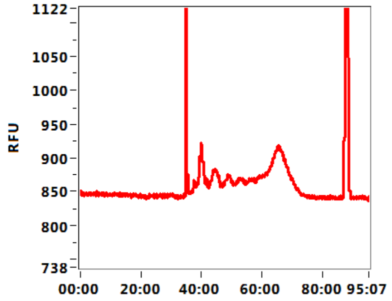

B6: cardiac B4 D7-3

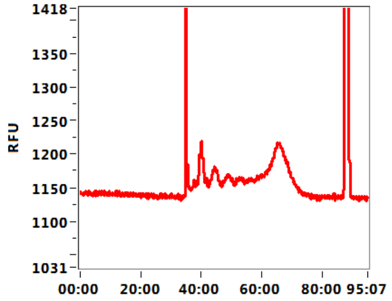

B7: cardiac B4 D9-1

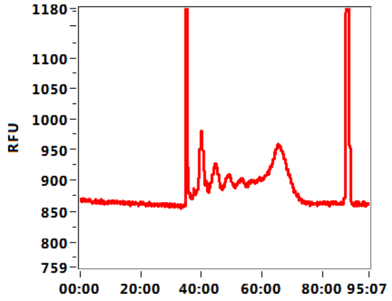

B8: cardiac B4 D9-2

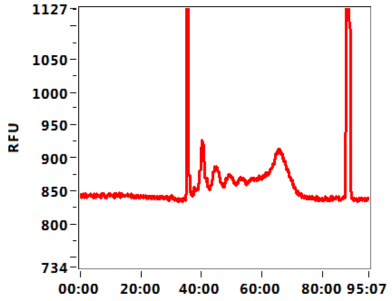

B9: cardiac B4 D9-3

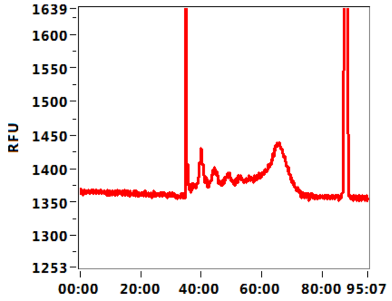

B10: cardiac B4 D12-1

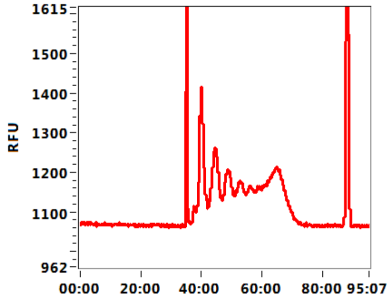

B11: cardiac B4 D12-2

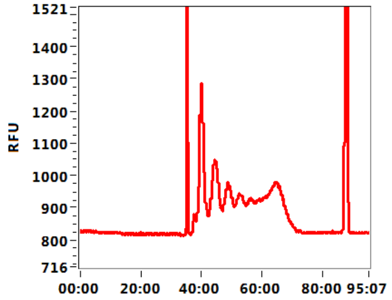

B12: cardiac B4 D12-3

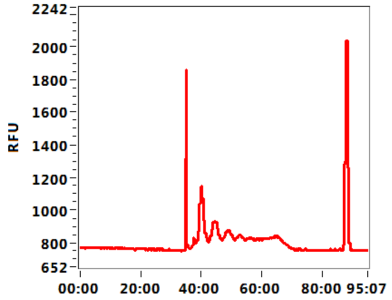

 Copyright 2015 Advanced Analytical Technologies, Inc.

Sample: cardiac B4 D6-1
Well Location: B1
Created: Wednesday, July 21, 2021 5:26:30 PM

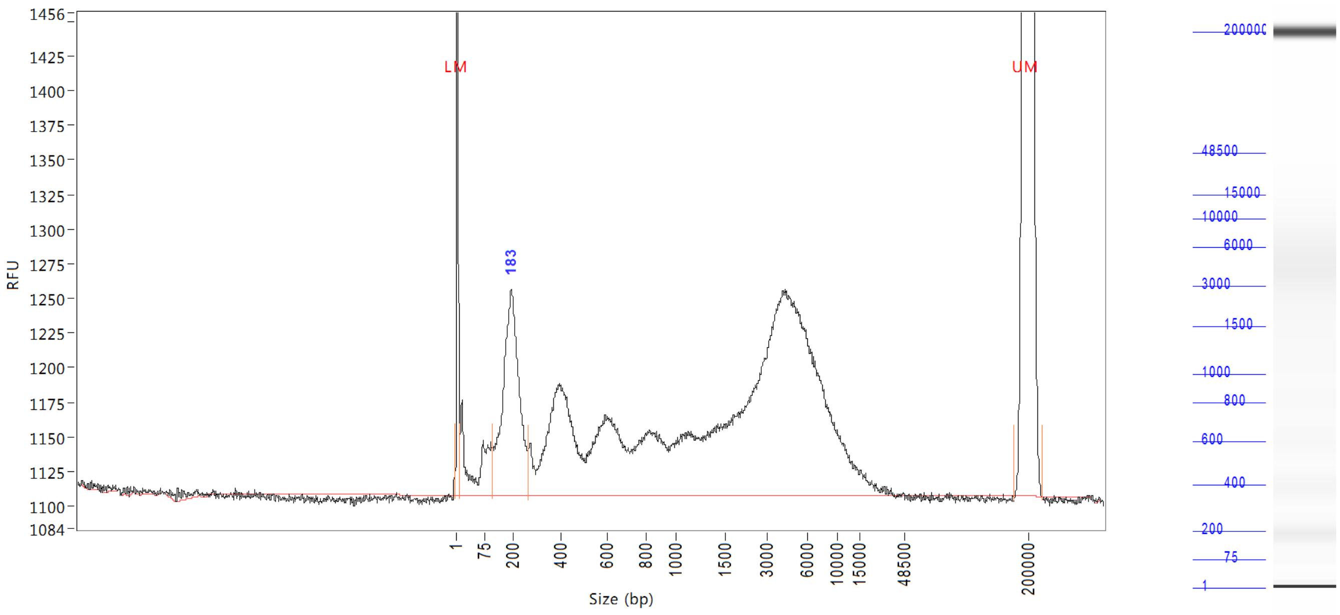

Peak	Size (bp)	Rel. Conc. %
1	1 (LM)	
2	183	100.0
3	200000 (UM)	

TIC:	0.1272	ng/uL
TIM:	1.142	nmole/L
Total Conc.:	0.7876	ng/uL

Sample Peak Width (sec): 10 Sample Min Peak Height: 50 Sample Baseline V to V?: Y Sample Baseline V to V pts: 3
Sample Filter: Binomial # of Pts for Filter: 3 Sample Start Region (min): 0 Sample End Region (min): 95
Manual Baseline Start (min): 12 Manual Baseline End (min): 94
Marker Peak Width (sec): 5 Marker Min Peak Height: 500 Marker Baseline V to V?: N Marker Baseline V to V pts: 3
Lower Marker Selection: First Peak > 500 RFU Upper Marker Selection: Last Peak > 500 RFU
Ladder Size (bp): 1, 75, 200, 400, 600, 800, 1000, 1500, 3000, 6000, 10000, 15000, 48500, 200000
Quantification Using: Ladder Final Concentration (ng/uL): 0.1250 Dilution Factor: 12.0
Min. RFU for Data Processing: 3

 Copyright 2015 Advanced Analytical Technologies, Inc.

Sample: cardiac B4 D6-2
Well Location: B2
Created: Wednesday, July 21, 2021 5:26:30 PM

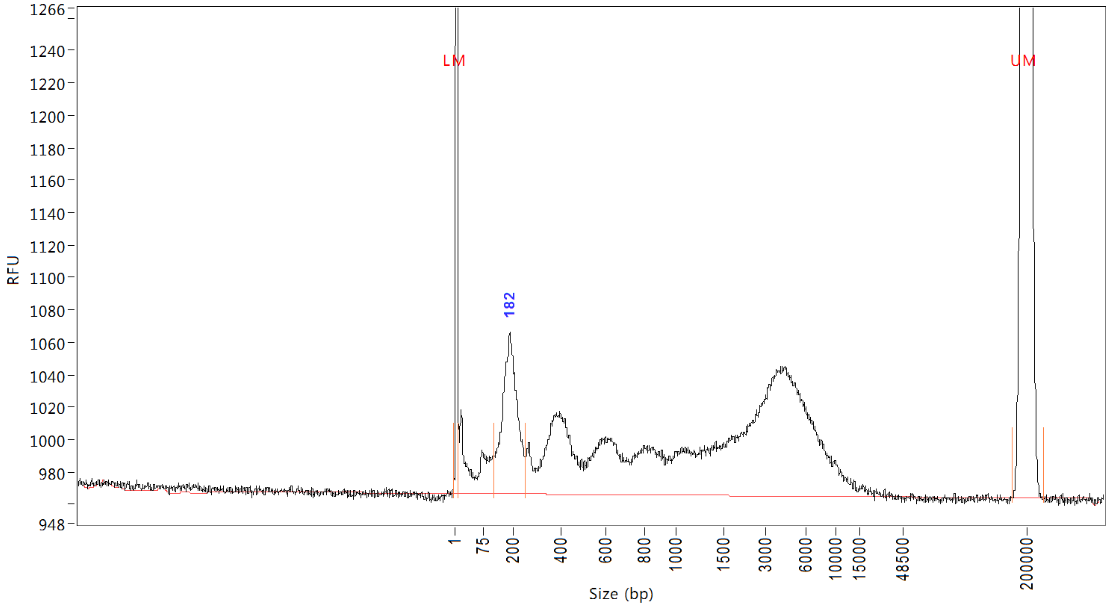

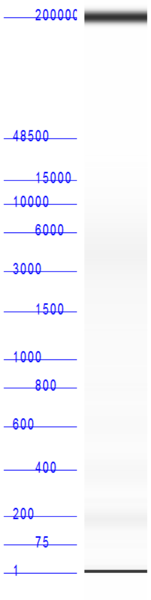

Peak	Size (bp)	Rel. Conc. %
1	1 (LM)	
2	182	100.0
3	200000 (UM)	

TIC:	0.1007	ng/uL
TIM:	0.912	nmole/L
Total Conc.:	0.5976	ng/uL

Sample Peak Width (sec): 10 Sample Min Peak Height: 50 Sample Baseline V to V?: Y Sample Baseline V to V pts: 3
Sample Filter: Binomial # of Pts for Filter: 3 Sample Start Region (min): 0 Sample End Region (min): 95
Manual Baseline Start (min): 12 Manual Baseline End (min): 94
Marker Peak Width (sec): 5 Marker Min Peak Height: 500 Marker Baseline V to V?: N Marker Baseline V to V pts: 3
Lower Marker Selection: First Peak > 500 RFU Upper Marker Selection: Last Peak > 500 RFU
Ladder Size (bp): 1, 75, 200, 400, 600, 800, 1000, 1500, 3000, 6000, 10000, 15000, 48500, 200000
Quantification Using: Ladder Final Concentration (ng/uL): 0.1250 Dilution Factor: 12.0
Min. RFU for Data Processing: 3

 Copyright 2015 Advanced Analytical Technologies, Inc.

Sample: cardiac B4 D6-3
Well Location: B3
Created: Wednesday, July 21, 2021 5:26:30 PM

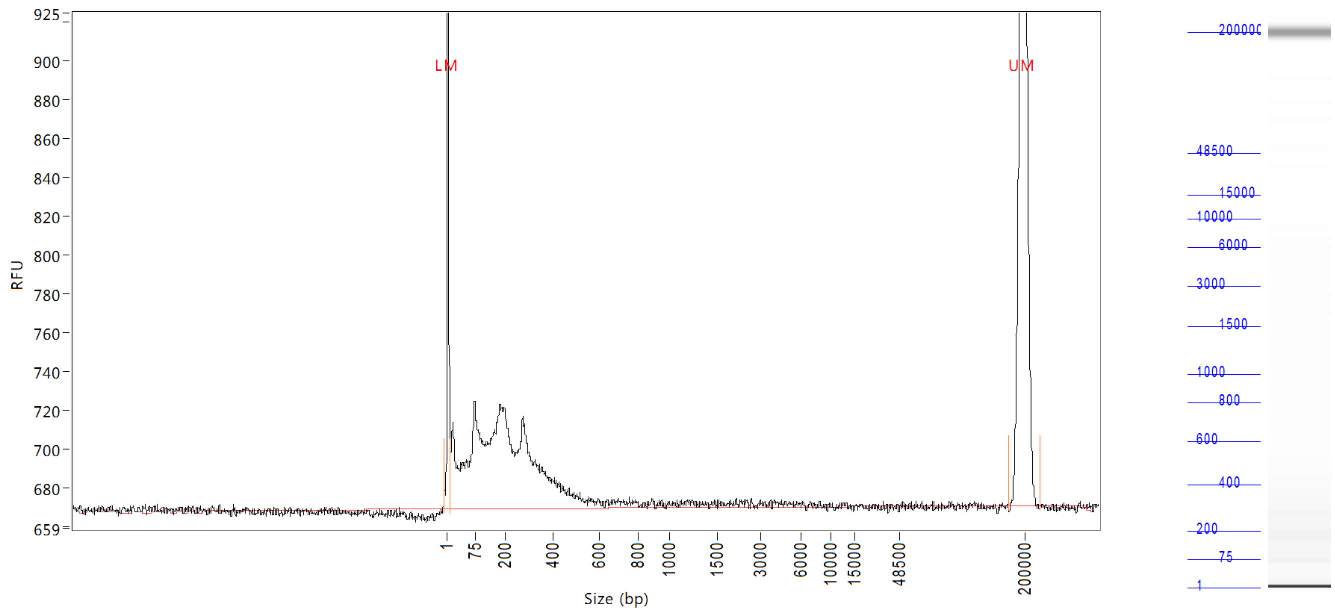

Peak	Size (bp)	Rel. Conc. %	
1	1 (LM)		
2	200000 (UM)		
	TIC:	0.0000	ng/uL
	TIM:	0.000	nmole/L
	Total Conc.:	0.2725	ng/uL

Sample Peak Width (sec): 10   Sample Min Peak Height: 50   Sample Baseline V to V?: Y   Sample Baseline V to V pts: 3
Sample Filter: Binomial   # of Pts for Filter: 3   Sample Start Region (min): 0   Sample End Region (min): 95
Manual Baseline Start (min): 12   Manual Baseline End (min): 94
Marker Peak Width (sec): 5   Marker Min Peak Height: 500   Marker Baseline V to V?: N   Marker Baseline V to V pts: 3
Lower Marker Selection: First Peak > 500 RFU   Upper Marker Selection: Last Peak > 500 RFU
Ladder Size (bp): 1, 75, 200, 400, 600, 800, 1000, 1500, 3000, 6000, 10000, 15000, 48500, 200000
Quantification Using: Ladder   Final Concentration (ng/uL): 0.1250   Dilution Factor: 12.0
Min. RFU for Data Processing: 3

 Copyright 2015 Advanced Analytical Technologies, Inc.

Sample: cardiac B4 D7-1
Well Location: B4
Created: Wednesday, July 21, 2021 5:26:30 PM

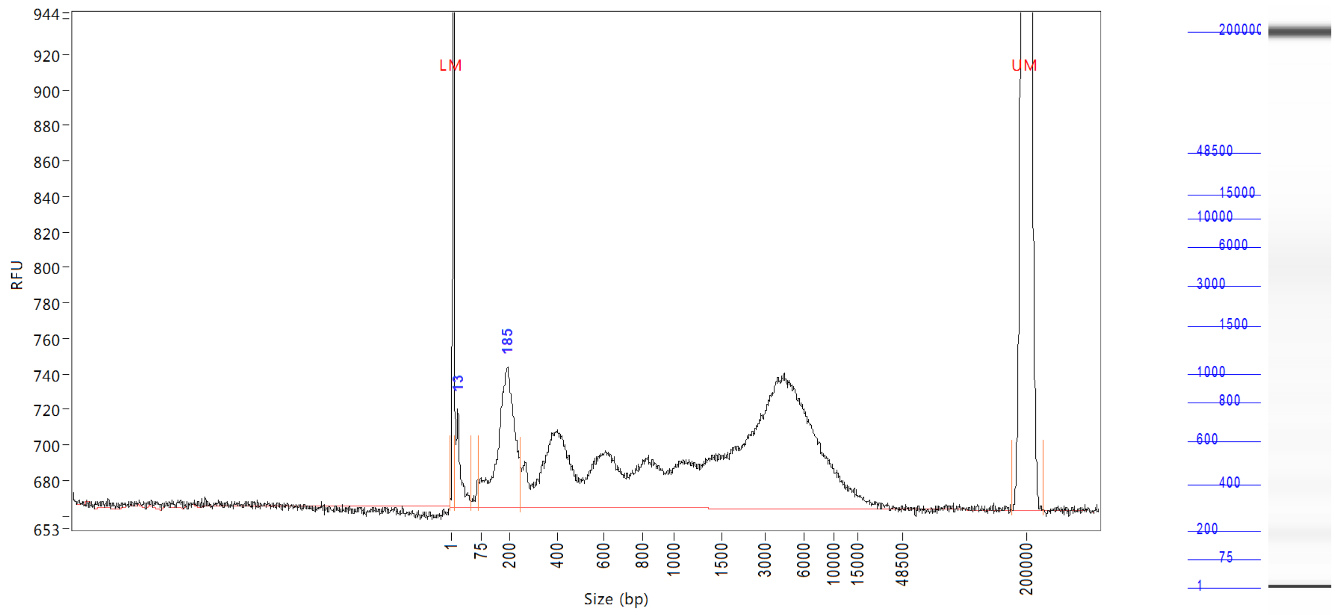

Peak	Size (bp)	Rel. Conc. %
1	1 (LM)	
2	13	15.1
3	185	84.9
4	200000 (UM)	

TIC:	0.1175	ng/uL
TIM:	3.051	nmole/L
Total Conc.:	0.5875	ng/uL

```
Sample Peak Width (sec): 10   Sample Min Peak Height: 50    Sample Baseline V to V?: Y    Sample Baseline V to V pts: 3
Sample Filter: Binomial       # of Pts for Filter: 3        Sample Start Region (min): 0  Sample End Region (min): 95
Manual Baseline Start (min): 12     Manual Baseline End (min): 94
Marker Peak Width (sec): 5    Marker Min Peak Height: 500   Marker Baseline V to V?: N    Marker Baseline V to V pts: 3
Lower Marker Selection: First Peak > 500 RFU                Upper Marker Selection: Last Peak > 500 RFU
Ladder Size (bp): 1, 75, 200, 400, 600, 800, 1000, 1500, 3000, 6000, 10000, 15000, 48500, 200000
Quantification Using: Ladder        Final Concentration (ng/uL): 0.1250         Dilution Factor: 12.0
Min. RFU for Data Processing: 3
```

 Copyright 2015 Advanced Analytical Technologies, Inc.

Sample: cardiac B4 D7-2
Well Location: B5
Created: Wednesday, July 21, 2021 5:26:30 PM

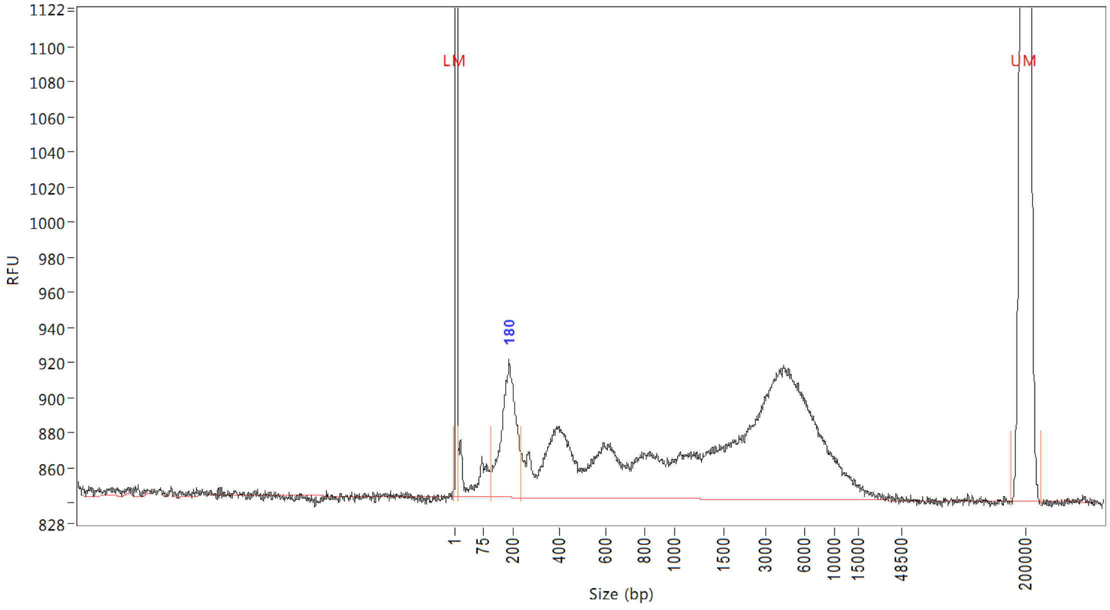

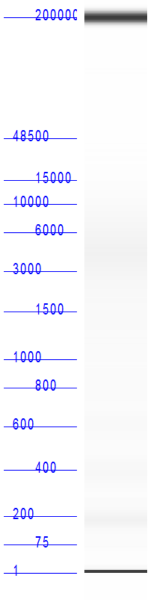

Peak	Size (bp)	Rel. Conc. %
1	1 (LM)	
2	180	100.0
3	200000 (UM)	

TIC:	0.0860	ng/uL
TIM:	0.785	nmole/L
Total Conc.:	0.5886	ng/uL

Sample Peak Width (sec): 10   Sample Min Peak Height: 50   Sample Baseline V to V?: Y   Sample Baseline V to V pts: 3
Sample Filter: Binomial   # of Pts for Filter: 3   Sample Start Region (min): 0   Sample End Region (min): 95
Manual Baseline Start (min): 12   Manual Baseline End (min): 94
Marker Peak Width (sec): 5   Marker Min Peak Height: 500   Marker Baseline V to V?: N   Marker Baseline V to V pts: 3
Lower Marker Selection: First Peak > 500 RFU   Upper Marker Selection: Last Peak > 500 RFU
Ladder Size (bp): 1, 75, 200, 400, 600, 800, 1000, 1500, 3000, 6000, 10000, 15000, 48500, 200000
Quantification Using: Ladder   Final Concentration (ng/uL): 0.1250   Dilution Factor: 12.0
Min. RFU for Data Processing: 3

 Copyright 2015 Advanced Analytical Technologies, Inc.

Sample: cardiac B4 D7-3
Well Location: B6
Created: Wednesday, July 21, 2021 5:26:30 PM

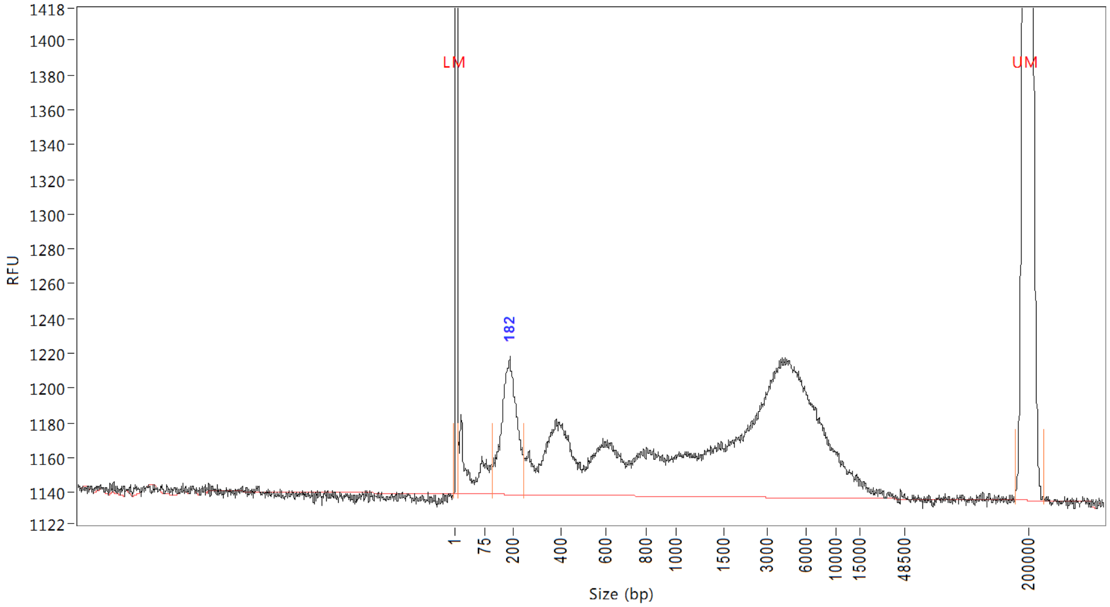

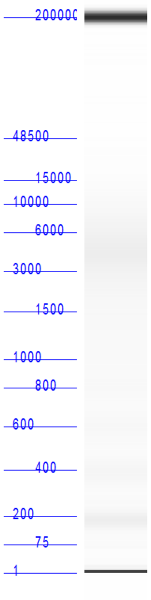

Peak	Size (bp)	Rel. Conc. %
1	1 (LM)	
2	182	100.0
3	200000 (UM)	

TIC:	0.1157	ng/uL
TIM:	1.047	nmole/L
Total Conc.:	0.7655	ng/uL

```
Sample Peak Width (sec): 10   Sample Min Peak Height: 50    Sample Baseline V to V?: Y    Sample Baseline V to V pts: 3
Sample Filter: Binomial       # of Pts for Filter: 3        Sample Start Region (min): 0  Sample End Region (min): 95
Manual Baseline Start (min): 12     Manual Baseline End (min): 94
Marker Peak Width (sec): 5    Marker Min Peak Height: 500   Marker Baseline V to V?: N    Marker Baseline V to V pts: 3
Lower Marker Selection: First Peak > 500 RFU                Upper Marker Selection: Last Peak > 500 RFU
Ladder Size (bp): 1, 75, 200, 400, 600, 800, 1000, 1500, 3000, 6000, 10000, 15000, 48500, 200000
Quantification Using: Ladder          Final Concentration (ng/uL): 0.1250          Dilution Factor: 12.0
Min. RFU for Data Processing: 3
```


Copyright 2015 Advanced Analytical Technologies, Inc.

Sample: cardiac B4 D9-1
Well Location: B7
Created: Wednesday, July 21, 2021 5:26:30 PM

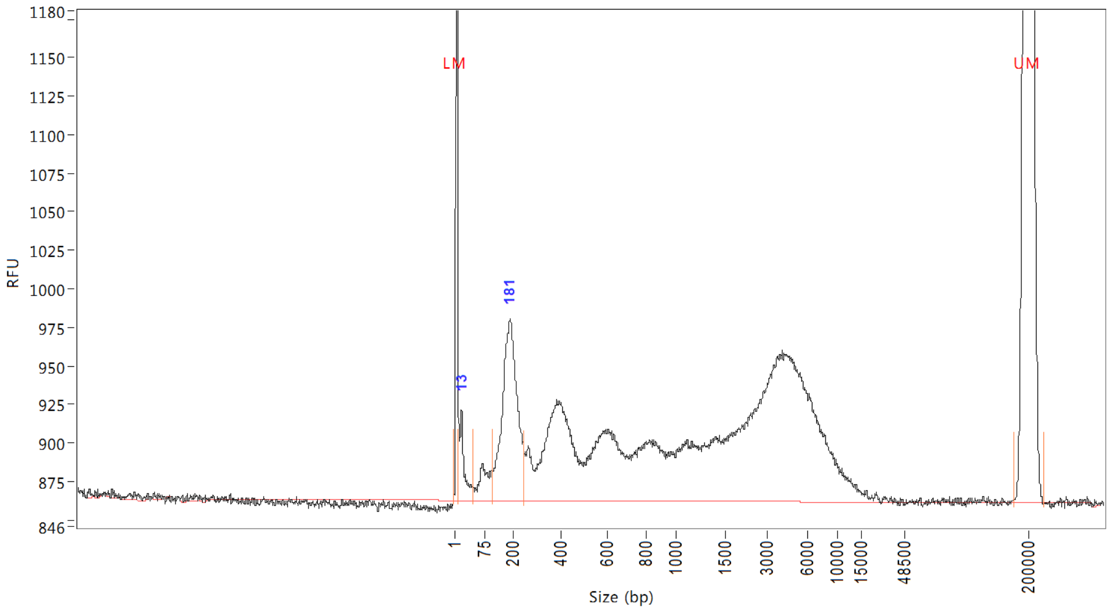

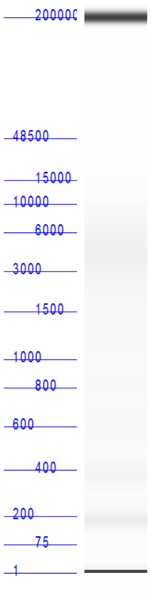

Peak	Size (bp)	Rel. Conc. %
1	1 (LM)	
2	13	13.4
3	181	86.6
4	200000 (UM)	

TIC:	0.1398	ng/uL
TIM:	3.390	nmole/L
Total Conc.:	0.7525	ng/uL

Sample Peak Width (sec): 10   Sample Min Peak Height: 50   Sample Baseline V to V?: Y   Sample Baseline V to V pts: 3
Sample Filter: Binomial   # of Pts for Filter: 3   Sample Start Region (min): 0   Sample End Region (min): 95
Manual Baseline Start (min): 12   Manual Baseline End (min): 94
Marker Peak Width (sec): 5   Marker Min Peak Height: 500   Marker Baseline V to V?: N   Marker Baseline V to V pts: 3
Lower Marker Selection: First Peak > 500 RFU   Upper Marker Selection: Last Peak > 500 RFU
Ladder Size (bp): 1, 75, 200, 400, 600, 800, 1000, 1500, 3000, 6000, 10000, 15000, 48500, 200000
Quantification Using: Ladder   Final Concentration (ng/uL): 0.1250   Dilution Factor: 12.0
Min. RFU for Data Processing: 3

Copyright 2015 Advanced Analytical Technologies, Inc.

Sample: cardiac B4 D9-2
Well Location: B8
Created: Wednesday, July 21, 2021 5:26:30 PM

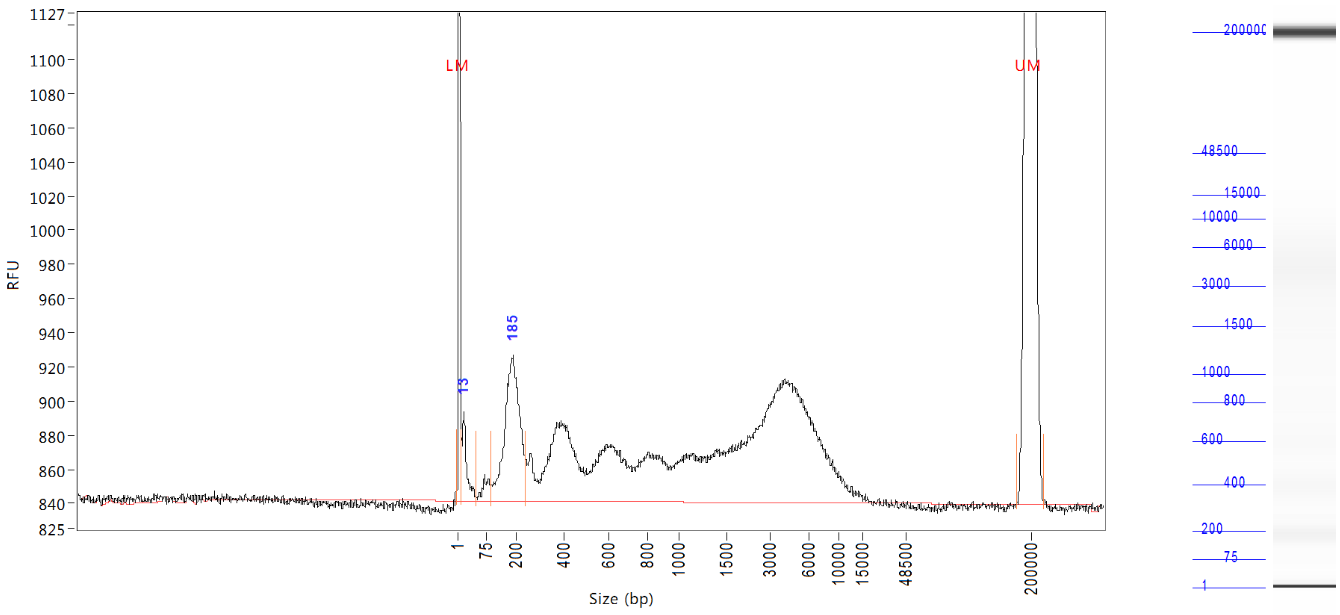

Peak	Size (bp)	Rel. Conc. %
1	1 (LM)	
2	13	14.4
3	185	85.6
4	200000 (UM)	

TIC:	0.1012	ng/uL
TIM:	2.605	nmole/L
Total Conc.:	0.5244	ng/uL

Sample Peak Width (sec): 10   Sample Min Peak Height: 50   Sample Baseline V to V?: Y   Sample Baseline V to V pts: 3
Sample Filter: Binomial   # of Pts for Filter: 3   Sample Start Region (min): 0   Sample End Region (min): 95
Manual Baseline Start (min): 12   Manual Baseline End (min): 94
Marker Peak Width (sec): 5   Marker Min Peak Height: 500   Marker Baseline V to V?: N   Marker Baseline V to V pts: 3
Lower Marker Selection: First Peak > 500 RFU   Upper Marker Selection: Last Peak > 500 RFU
Ladder Size (bp): 1, 75, 200, 400, 600, 800, 1000, 1500, 3000, 6000, 10000, 15000, 48500, 200000
Quantification Using: Ladder   Final Concentration (ng/uL): 0.1250   Dilution Factor: 12.0
Min. RFU for Data Processing: 3

 Copyright 2015 Advanced Analytical Technologies, Inc.

Sample: cardiac B4 D9-3
Well Location: B9
Created: Wednesday, July 21, 2021 5:26:30 PM

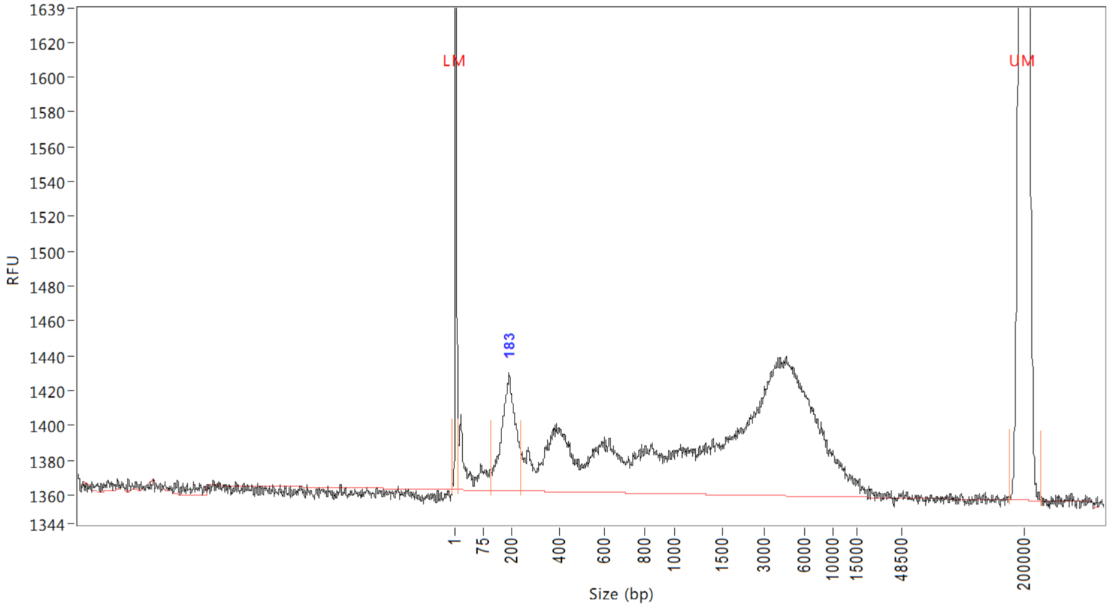

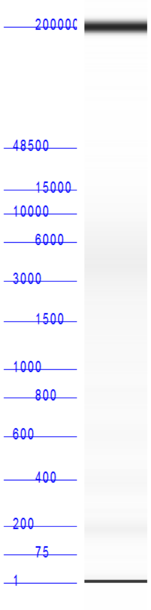

Peak	Size (bp)	Rel. Conc. %
1	1 (LM)	
2	183	100.0
3	200000 (UM)	

TIC:	0.0812	ng/uL
TIM:	0.729	nmole/L
Total Conc.:	0.6524	ng/uL

Sample Peak Width (sec): 10   Sample Min Peak Height: 50   Sample Baseline V to V?: Y   Sample Baseline V to V pts: 3
Sample Filter: Binomial   # of Pts for Filter: 3   Sample Start Region (min): 0   Sample End Region (min): 95
Manual Baseline Start (min): 12   Manual Baseline End (min): 94
Marker Peak Width (sec): 5   Marker Min Peak Height: 500   Marker Baseline V to V?: N   Marker Baseline V to V pts: 3
Lower Marker Selection: First Peak > 500 RFU   Upper Marker Selection: Last Peak > 500 RFU
Ladder Size (bp): 1, 75, 200, 400, 600, 800, 1000, 1500, 3000, 6000, 10000, 15000, 48500, 200000
Quantification Using: Ladder   Final Concentration (ng/uL): 0.1250   Dilution Factor: 12.0
Min. RFU for Data Processing: 3

 Copyright 2015 Advanced Analytical Technologies, Inc.

Sample: cardiac B4 D12-1
Well Location: B10
Created: Wednesday, July 21, 2021 5:26:30 PM

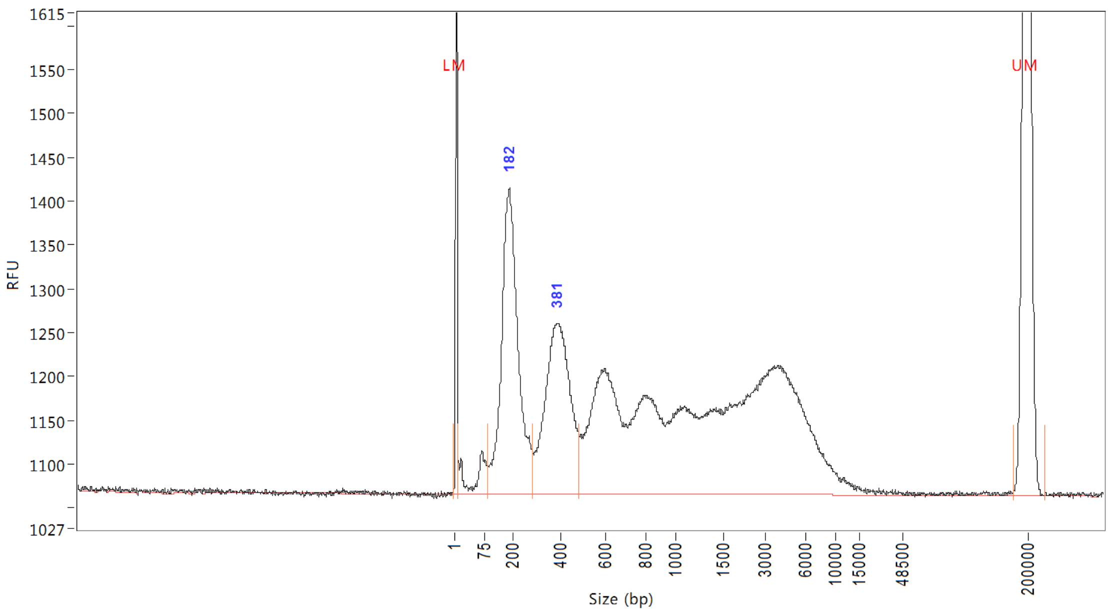

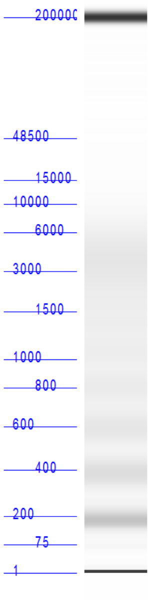

Peak	Size (bp)	Rel. Conc. %
1	1 (LM)	
2	182	56.7
3	381	43.3
4	200000 (UM)	

TIC:	0.8229	ng/uL
TIM:	5.742	nmole/L
Total Conc.:	2.0927	ng/uL

Sample Peak Width (sec): 10 Sample Min Peak Height: 50 Sample Baseline V to V?: Y Sample Baseline V to V pts: 3
Sample Filter: Binomial # of Pts for Filter: 3 Sample Start Region (min): 0 Sample End Region (min): 95
Manual Baseline Start (min): 12 Manual Baseline End (min): 94
Marker Peak Width (sec): 5 Marker Min Peak Height: 500 Marker Baseline V to V?: N Marker Baseline V to V pts: 3
Lower Marker Selection: First Peak > 500 RFU Upper Marker Selection: Last Peak > 500 RFU
Ladder Size (bp): 1, 75, 200, 400, 600, 800, 1000, 1500, 3000, 6000, 10000, 15000, 48500, 200000
Quantification Using: Ladder Final Concentration (ng/uL): 0.1250 Dilution Factor: 12.0
Min. RFU for Data Processing: 3

 Copyright 2015 Advanced Analytical Technologies, Inc.

Sample: cardiac B4 D12-2
Well Location: B11
Created: Wednesday, July 21, 2021 5:26:30 PM

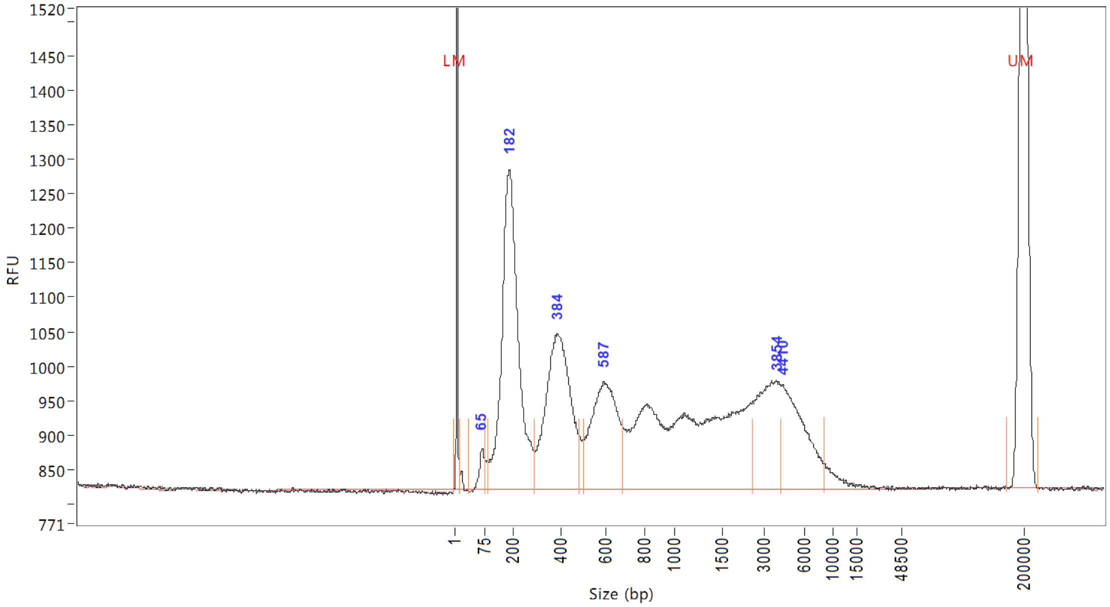

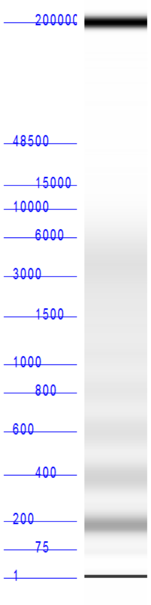

Peak	Size (bp)	Rel. Conc. %
1	1 (LM)	
2	65	1.5
3	182	36.9
4	384	24.6
5	587	15.9
6	3854	11.1
7	4410	10.0
8	200000 (UM)	

TIC:	1.8742	ng/uL
TIM:	9.952	nmole/L
Total Conc.:	2.6515	ng/uL

```
Sample Peak Width (sec): 10   Sample Min Peak Height: 50    Sample Baseline V to V?: Y    Sample Baseline V to V pts: 3
Sample Filter: Binomial       # of Pts for Filter: 3        Sample Start Region (min): 0  Sample End Region (min): 95
Manual Baseline Start (min): 12     Manual Baseline End (min): 94
Marker Peak Width (sec): 5    Marker Min Peak Height: 500   Marker Baseline V to V?: N    Marker Baseline V to V pts: 3
Lower Marker Selection: First Peak > 500 RFU                Upper Marker Selection: Last Peak > 500 RFU
Ladder Size (bp): 1, 75, 200, 400, 600, 800, 1000, 1500, 3000, 6000, 10000, 15000, 48500, 200000
Quantification Using: Ladder          Final Concentration (ng/uL): 0.1250          Dilution Factor: 12.0
Min. RFU for Data Processing: 3
```

 Copyright 2015 Advanced Analytical Technologies, Inc.

Sample: cardiac B4 D12-3
Well Location: B12
Created: Wednesday, July 21, 2021 5:26:30 PM

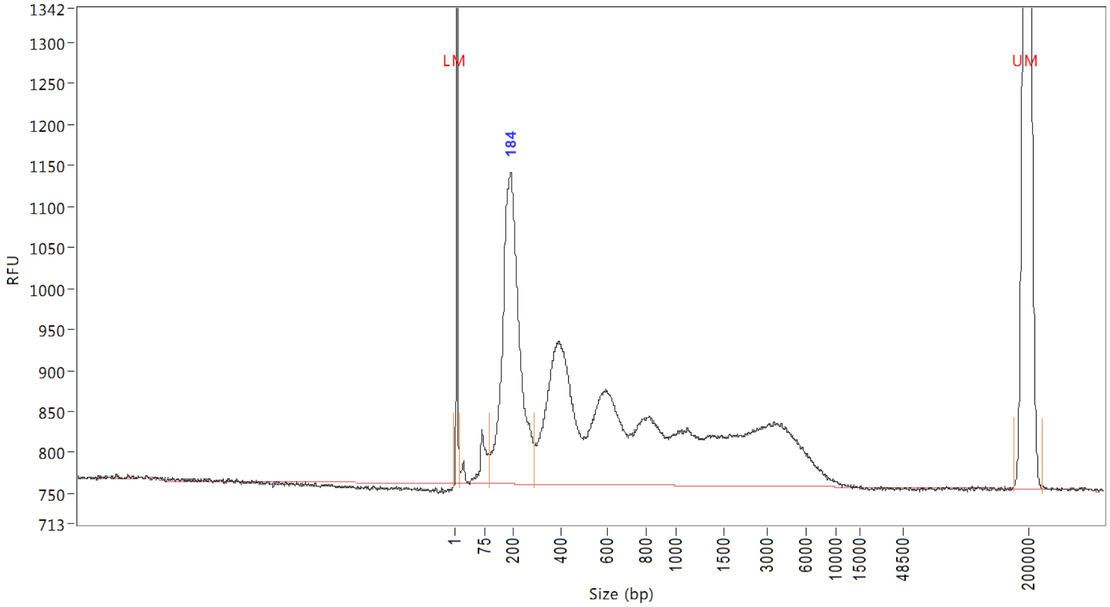

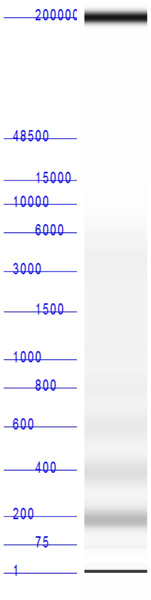

Peak	Size (bp)	Rel. Conc. %
1	1 (LM)	
2	184	100.0
3	200000 (UM)	

TIC:	0.5297	ng/uL
TIM:	4.736	nmole/L
Total Conc.:	1.6995	ng/uL

```
Sample Peak Width (sec): 10   Sample Min Peak Height: 200   Sample Baseline V to V?: Y    Sample Baseline V to V pts: 3
Sample Filter: Binomial       # of Pts for Filter: 3        Sample Start Region (min): 0  Sample End Region (min): 95
Manual Baseline Start (min): 12     Manual Baseline End (min): 94
Marker Peak Width (sec): 5    Marker Min Peak Height: 500   Marker Baseline V to V?: N    Marker Baseline V to V pts: 3
Lower Marker Selection: First Peak > 500 RFU                Upper Marker Selection: Last Peak > 500 RFU
Ladder Size (bp): 1, 75, 200, 400, 600, 800, 1000, 1500, 3000, 6000, 10000, 15000, 48500, 200000
Quantification Using: Ladder          Final Concentration (ng/uL): 0.1250          Dilution Factor: 12.0
Min. RFU for Data Processing: 3
```

Sample: DNA Size Ladder
Well Location: A12
Created: Wednesday, July 21, 2021 5:26:30 PM
Import From: C:\Users\aati\Desktop\cfDNA ladder.SCAL
Fit Type: Point to Point

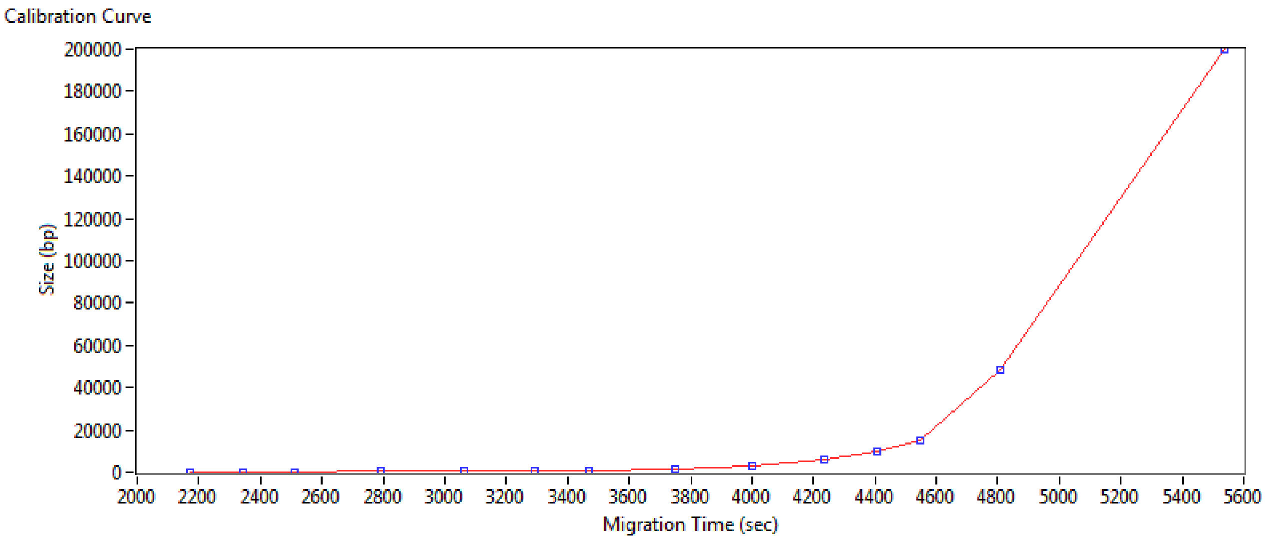

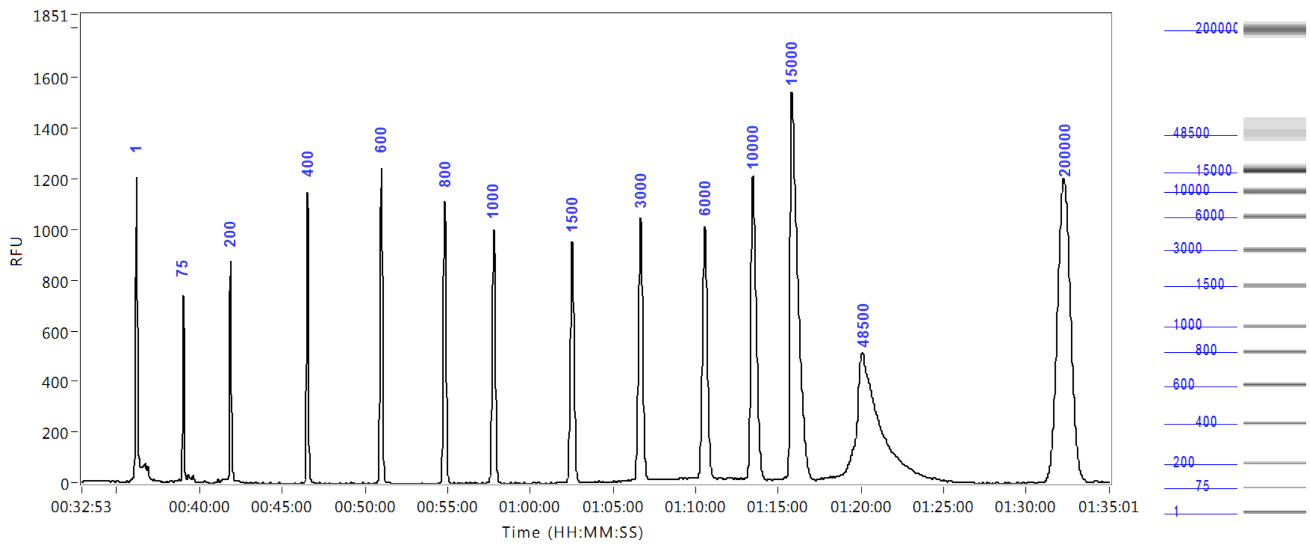

Copyright 2015 Advanced Analytical Technologies, Inc.
